# PAX5 modulates vascularization and contributes to neuroendocrine lineage transition during carcinoma progression

**DOI:** 10.1172/JCI190596

**Published:** 2026-07-23

**Authors:** Ailing Wu, Yujie Hao, Xuemiao Yan, Junrong Liu, Lin Wang, Yan Jin, Wenxu Liu, Xiyue Chen, Yuan Jiang, Luc Girard, Zhiqun Shang, Jun Yan, Zhenfa Zhang, Wenchen Gong, Yuanjie Niu, Benjamin J. Drapkin, John D. Minna, Lance S. Terada, Zhenyi Ma, Zhe Liu

**Affiliations:** 1 Zhejiang Key Laboratory of Medical Epigenetics, Jiande Hospital, Department of Cell Biology, School of Basic Medical Sciences, Hangzhou Normal University, Hangzhou, China.; 2 Department of Pathology, Tianjin First Center Hospital, Tianjin, China.; 3 Department of Lung Cancer Center, Tianjin Medical University Cancer Institute and Hospital, Tianjin, China.; 4 Hamon Center for Therapeutic Oncology Research, University of Texas Southwestern Medical Center, Dallas, Texas, USA.; 5 Tianjin Institute of Urology, the 2nd Hospital of Tianjin Medical University, Tianjin, China.; 6 Department of Pathology, Tianjin First Center Hospital, Tianjin, China.; 7 Department of Pathology, Tianjin Medical University Cancer Institute and Hospital, Tianjin, China.; 8 Department of Internal Medicine and Simmons Comprehensive Cancer Center and; 9 Department of Internal Medicine, Division of Pulmonary and Critical Care, University of Texas Southwestern Medical Center, Dallas, Texas, USA.; 10 Collaborative Innovation Center for Cancer Personalized Medicine, Nanjing Medical University, Nanjing, China.

**Keywords:** Angiogenesis, Oncology, Lung cancer, Mouse models, Neuroendocrine regulation

## Abstract

Human carcinomas often gain aggressive characteristics and escape cell type–specific treatment regimens through cryptic shifts in lineage states. However, the underlying mechanisms that govern lineage plasticity in carcinomas are undefined. Here in this study, we found that *PAX5*, a neural/lymphatic transcription factor, contributed to neuroendocrine (NE) lineage transition. *PAX5* was highly expressed in aggressive human NE carcinoma cells and tissues but not in non-NE cancer cells and tissues. Deletion of *Pax5* in *Rb1^fl/fl^ Trp53^fl/fl^* mice caused a reduction of tumor vessels, loss of NE morphologic features, and decreased expression of ASCL1, NCAM, and SYP, whereas ectopic expression of *PAX5* in CC10-rtTA TetO-*hEGFR*^ex19del/T790M^ mouse adenocarcinomas and in LNCAP prostate cancer xenografts induced an angiogenic microenvironment and NE morphology. Importantly, antiangiogenic drugs reduced NE features of *Rb1^fl/fl^ Trp53^fl/fl^* tumors and blocked *PAX5*-induced NE transformation. These studies demonstrate an essential role of an angiogenic microenvironment in transition/maintenance of NE lineage, suggesting that targeting *PAX5* and its downstream signaling may modulate lineage transitions responsible for treatment failure in both small cell neuroendocrine carcinoma and adenocarcinomas.

## Abstract 2

PAX5 generates angiogenic microenvironment and drives neuroendocrine transition in aggressive carcinomas. Targeing angiogenesis may imporve outcome by preventing neuroendocrine transition.

## Graphical abstract

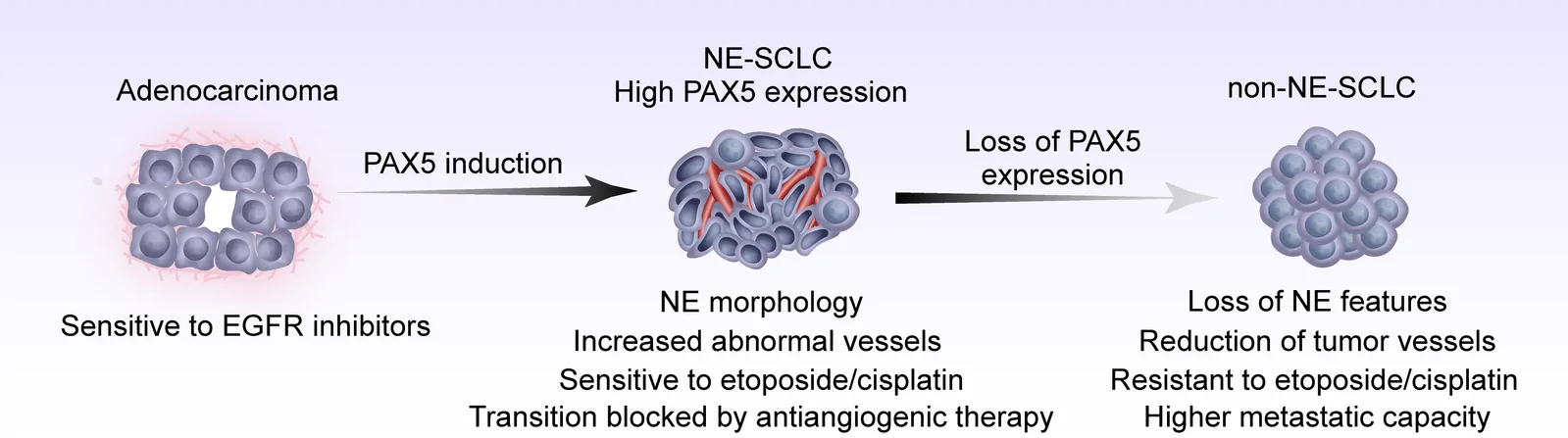

## Introduction

Small cell neuroendocrine carcinoma (SCNC) is a highly aggressive, lethal, and widely metastatic cancer that can originate from almost all epithelial organs, including the lung (small cell lung cancer, SCLC) and prostate (small cell prostate cancer, SCPC). Primary SCLC accounts for 15% of lung cancers, whereas primary SCPC is rare and accounts for less than 1% of prostate cancers. SCNCs exhibit pathological and biological characteristics highly distinct from other epithelial cancers: they are smaller, have shorter doubling time, express a neuroendocrine program, exhibit almost universal inactivation of *TP53* and *RB1*, and display frequent disruption of Notch, TGF-β, and Hippo pathways (1–4). Most SCNCs are initially responsive to standard systemic therapy (platinum-based chemotherapy with etoposide) but rapidly develop resistance and relapse with higher metastatic capability. For decades, meaningful progress in SCNC treatment has been hampered by limited understanding of the molecular mechanisms driving this malignancy and its escape from chemosensitivity, leading to its designation as a “recalcitrant cancer.” Of importance, relapsed small cell carcinomas often lose neuroendocrine (NE) and gain non-NE features as they acquire resistance to chemotherapeutic agents (5–12). The mechanism responsible for this transition from NE to non-NE states is completely unknown.

In addition to this NE to non-NE transition seen in SCNCs, transformation to SCNCs is seen in primary adenocarcinomas. A substantial subset of lung (10%) and prostate (17%) adenocarcinomas transform into SCNC tumors when given potent inhibitors of active oncogenic pathways, developing treatment resistance to these drugs (13–16). Whole-genome sequencing of resistant tumors that either maintained adenocarcinoma or transformed into SCNC revealed that *RB1* inactivation is associated with adenocarcinoma-to-SCNC transformation in both lung and prostate (17). However, the epigenetic shift and the associated transcription factors (TFs) driving this conversion are undefined.

Here, we generated a single-cell transcriptomic map of human primary SCLC and found that primary SCLC tissues harbor a minor subset of non–NE-SCLC cells even before treatment. By focusing on TFs differentially active in NE-SCLC cells compared with non–NE-SCLC cells, we identified *PAX5*, which controls both neural system and lymphocyte development, as a molecular determinant of NE lineage identity in carcinomas independent of tissue of origin. Our results additionally suggest therapeutic strategies for both NE and PAX5-positive adenocarcinomas that may prevent lineage transition during cell type–specific treatment.

## Results

### Single-cell RNA sequencing reveals the presence of non–NE-SCLC cells in human primary SCLC tissues.

**Figure 1:**
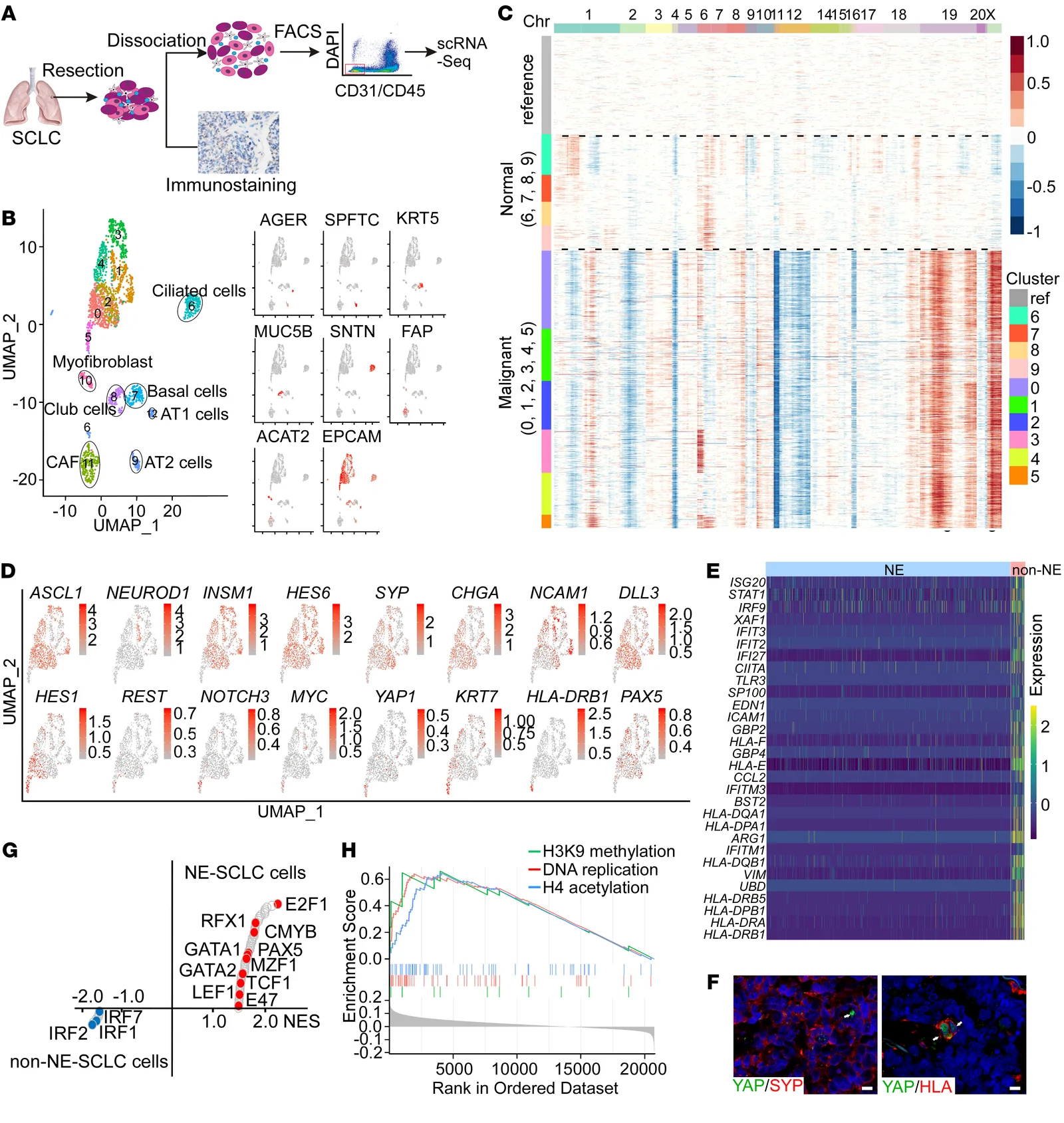
Single-cell RNA sequencing reveals the presence of non–NE-SCLC cells in human primary SCLC tissues. (**A**) Workflow depicts rapid dissociation and isolation of SCLC cells from primary tumors for generating single-cell RNA sequencing (scRNA-seq). (**B**) UMAP analysis shows transcriptionally distinct clusters (left panel). Right panels show expression of known markers. (**C**) CNV profiles inferred from scRNA-seq. (**D**) Expression of indicated genes. (**E**) Heatmap shows differentially expressed genes involved in interferon signaling. (**F**) Co-immunofluorescence of indicated proteins. Scale bar, 20 μm. (**G**) Enriched transcription factor binding motifs in NE-SCLC cells versus non–NE-SCLC cells identified by GSEA from the MSigDB database (geneset ID: C3: TFT) using the software provided by the Broad Institute. (**H**) GSEA shows the top 3 enriched pathways positively correlated with *PAX5* expression. CAF, cancer-associated fibroblasts; AT1, alveolar type 1 cells; AT2, alveolar type 2 cells.

To study intratumoral heterogeneity, we isolated individual cells from a freshly resected and dissociated human primary SCLC (Supplemental Table 1; supplemental material available online with this article; https://doi.org/10.1172/JCI190596DS1), removed inflammatory infiltrate and endothelial cells, and performed single-cell analysis using the 10x Genomics Chromium platform (Figure 1A). A total of 2,001 cells passed quality controls and were retained for downstream analysis (Supplemental Figure 1, A–C). We grouped cells according to expression profiles using uniform manifold approximation and projection (UMAP) analysis, and we identified 12 transcriptionally distinct cell clusters (Figure 1B). We distinguished malignant (cluster 0, 1, 2, 3, 4, 5) from nonmalignant cells (cluster 6, 7, 8, 9, 10, 11) by gene expression characteristics and by inferred copy number variation (CNV) analysis using published human normal lung epithelial cells as a control (Figure 1, B and C, and Methods).

Clusters 0, 1, 2, 3, and 4 were clustered mainly by cell cycle–related genes (Supplemental Figure 1D); expressed at least 1 of the NE lineage master TFs *ASCL1* or *NEUROD1*, NE markers (*INSM1*, *HES6*, *SYP*, *CHGA*, *NCAM1*), and the Notch signaling inhibitor *DLL3*; and were classified as NE-SCLC cells. However, cluster 5 (accounting for 4.7% of total malignant cells) lost expression of most NE markers but expressed *KRT7*, *MYC*, Notch signaling pathway genes (*HES1*, *REST*, *NOTCH3*), and Hippo pathway gene *YAP1* and were classified as non–NE-SCLC cells (Figure 1D). In addition, non–NE-SCLC cells exhibited induction of interferon signaling pathway genes and expressed HLA class II molecules (Figure 1, D and E). The transcriptional patterns of non–NE-SCLC cells resemble those of SCLC variant lines derived from post-therapy tumors that had recurred and cisplatin-resistant SCLC tumors (18). We confirmed the presence of HLA class II–expressing/YAP-expressing non–NE-SCLC cells in the corresponding primary SCLC tissue (Figure 1F). The transcriptional characteristics of different clusters are consistent with those of SCLC subtypes reported previously (6, 8, 18–22). The coexistence of different subtypes in one single SCLC tumor is in accordance with the high plasticity of SCLC.

### PAX5 expression is associated with NE phenotype.

We next explored key TFs that drive the transition from NE-SCLCs to non–NE-SCLCs. TF binding motif enrichment analysis identified 59 TFs differentially active in NE-SCLC cells and 7 TFs differentially active in non–NE-SCLC cells. As expected, motifs of E2F1, a TF that regulates a set of cell cycle genes and can be activated by the loss of RB, was the top enriched motif in NE-SCLCs (Figure 1G). RB inactivation has been identified in up to 90% of SCLCs and is considered an initiating molecular event of SCLC (23). Four of 7 motifs enriched in non–NE-SCLC cells are bound by IRFs, confirming the enrichment analysis showing that the top upregulated pathways in non–NE-SCLC cells are interferon signaling pathways. Notably, among the 59 NE-SCLC–enriched TFs, 9 are involved in hematopoietic cell development. Of these, *PAX5* was noted to be preferentially expressed in NE-SCLC clusters 0–4 but not non–NE-SCLC cluster 5 (Figure 1D). PAX5 is of particular interest as a potential lineage gatekeeper for NE-SCLC for 2 reasons. First, PAX5 is normally expressed in developing neural cells and in B lymphocytes, promoting both neuroepithelial and lymphocyte development (24–27). Besides showing NE characteristics, SCNCs also share multiple similarities in histology, metastatic capacity, and drug sensitivity with aggressive lymphomas (28). Second, gene set enrichment analysis (GSEA) of our scRNA-seq data revealed that the top enriched pathways positively correlated with *PAX5* expression level were cell cycle–related DNA replication and H3K9 methylation, which marks heterochromatin (Figure 1H). This result was confirmed by GSEA of published scRNA-seq of SCLC circulating tumor cell–derived patient-derived xenografts (19) and published scRNA-seq of human primary SCLCs (29) (Supplemental Figure 1E). Since rapid proliferation and hyperchromatic nuclei are 2 characteristics distinguishing NE-SCLC from non–NE-SCLC, we explored the possibility that PAX5 might contribute to NE-SCLC differentiation.

**Figure 2:**
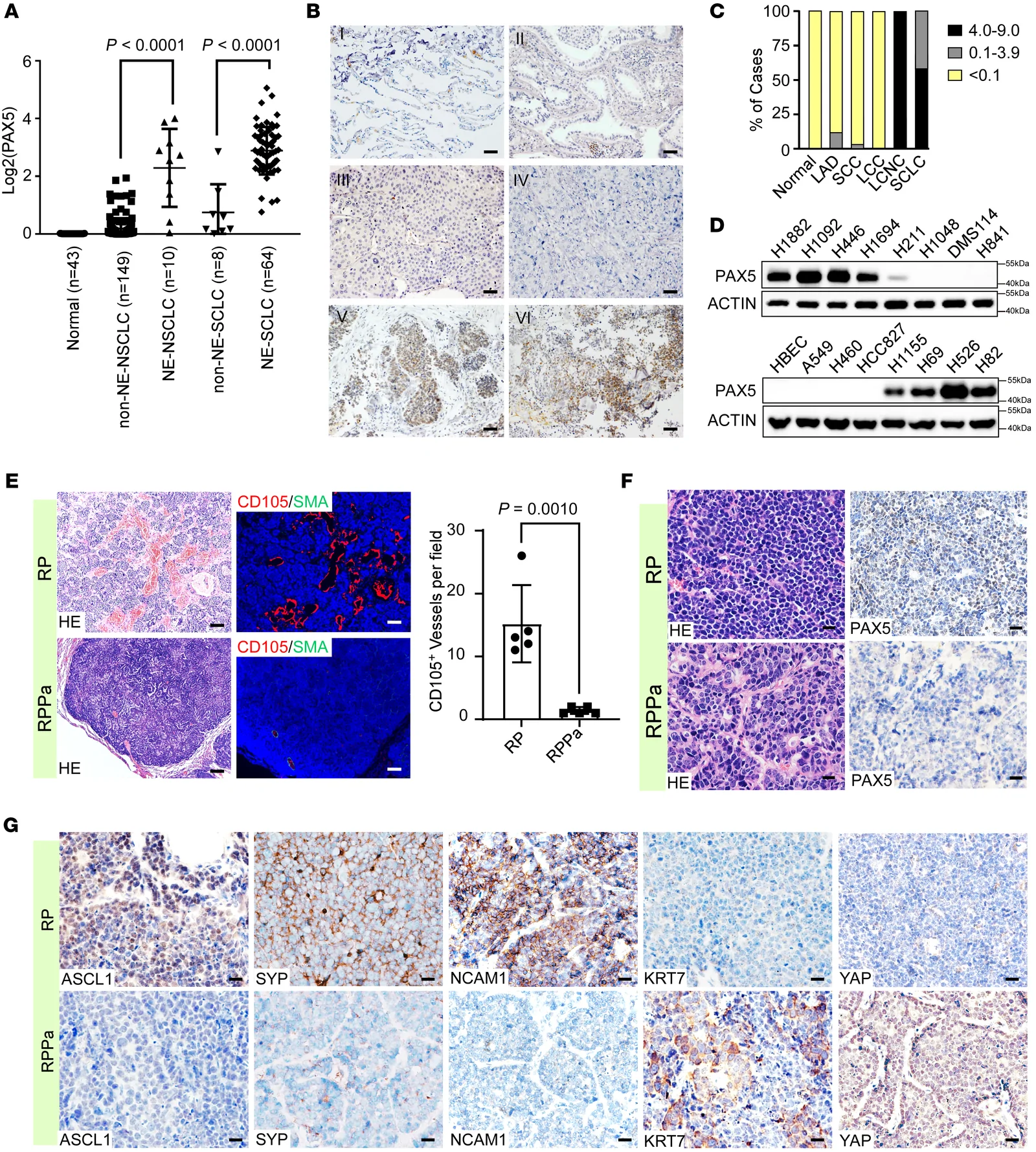
PAX5 maintains neuroendocrine features in SCLC. (**A**) Expression of *PAX5* was assessed. Log base 2 of the signal strength is shown. Data are presented as mean ± SD; 1-way ANOVA followed by Tukey’s multiple comparisons test for indicated pairwise comparisons. (**B**) Representative PAX5 immunostaining in sections of normal lung tissue (normal, I, *n* = 17), lung adenocarcinoma (LAD, II, *n* = 98), squamous cell carcinoma (SCC, III, *n* = 66), non-neuroendocrine large cell carcinoma (LCC, IV, *n* = 5), large cell neuroendocrine carcinoma (LCNC, V, *n* = 3), and small cell lung carcinoma (SCLC, VI, *n* = 37). Scale bar, 50 μm. (**C**) Scoring frequency of PAX5 immunostaining corresponding to the cases shown in **B**. Immunoreactivity was evaluated based primarily on nuclear expression. (**D**) Immunoblot shows expression of PAX5 and ACTIN in various SCLC cells. (**E**) Representative H&E staining and immunofluorescence images of CD105 and α-SMA in tumor sections from RP and RPPa mice (left panels, *n* = 5 independent mice per group), with the corresponding quantification of CD105^+^α-SMA^–^ vessels (right panel). Scale bar, 100 μm. Data are shown as mean ± SD (*n* = 5 mice per group); unpaired, 2-tailed Student’s *t* test. (**F** and **G**) H&E staining and immunostaining images of tumors developed in RP mice and RPPa mice. Scale bar, 20 μm.

We screened 274 human lung epithelial cell lines for expression of *PAX5* (30). Normal lung epithelium did not express *PAX5*. Non–NE–non–small cell lung cancer (non–NE-NSCLC), and non–NE-SCLC expressed significantly lower levels of *PAX5* compared with NE-NSCLC and NE-SCLC (Figure 2A). GSEA revealed that compared with *PAX5*-negative SCLC, *PAX5*-positive tumors were enriched in signaling pathways related to cell cycling and heterochromatin organization (Supplemental Figure 2A), suggesting that *PAX5*-positive tumors exhibit higher proliferation and condensed nuclear morphology, consistent with our single-cell data. At the protein level in human tissues, PAX5 was detected by immunohistochemistry in all SCLCs (37/37) and all NE-NSCLCs. In contrast, only 12 of 98 lung adenocarcinomas and 2 of 66 squamous cell carcinomas expressed intermediate levels of PAX5 (Figure 2, B and C). We next examined PAX5 expression in different subtypes of lung cancer cell lines. PAX5 was expressed in ASCL1^+^ SCLC lines (H1882, H69, H1092) and NEUROD1^+^ SCLC lines (H446, H1694, H82). In contrast, POU2F3^+^ SCLC lines (H211, H1048), with the exception of H526, as well as YAP1^+^ SCLC lines (DMS114, H841) showed no PAX5 expression (Figure 2D). These data indicate that PAX5 is predominantly expressed in NE lung cancer cells.

### PAX5 deletion causes loss of NE features.

To test the role of PAX5 in NE lung tumors, we crossed *Pax5^fl/fl^* mice (31) to *Rb1^fl/fl^ Trp53^fl/fl^* (RP) animals (32) to generate *Rb1^fl/fl^ Trp53^fl/fl^ Pax5^fl/fl^* (RPPa) mice (Supplemental Figure 2B). We intratracheally delivered adenoviruses carrying Cre driven by CMV promoter into RP mice, which develop tumors with SCLC phenotypes within 8 months (33), and RPPa mice. We induced 55 RP and 40 RPPa mice, and 49 RP and 35 RPPa mice developed tumors that we evaluated by histology examination. Three RP and 3 RPPa mice were examined 5 months after CMV-Cre inhalation, and all of them developed tumor nodules at large bronchioles (RP1: 5 tumor nodules, RP2: 9 tumor nodules, RP3: 5 tumor nodules, RPPa1, 6 tumor nodules, RPPa2: 10 tumor nodules, RPPa3: 7 tumor nodules). All of these early-stage tumor nodules exhibited similar histology and expressed ASCL1 and NCAM1 (Supplemental Figure 2, C and D). At 8 months of viral infection, both RP and RPPa mice began to exhibit labored breathing and harbored noticeable lung tumor nodules, liver metastatic nodules, or kidney metastatic nodules (Supplemental Figure 2E). Although the survival rates of RP and RPPa mice were similar (Supplemental Figure 2F), histological analysis of randomly selected late-stage RP (*n* = 5) and RPPa (*n* = 5) tumors revealed that RP and RPPa tumors exhibited marked morphological and pathological differences. First, tumors that developed in RP mice contained significantly more vessels than those that developed in RPPa mice. These vessels were positive for CD105, a protein expressed in proliferating angiogenic endothelial cells (34, 35), but were negative for α–smooth muscle actin (α-SMA), which marks vascular mural cells, indicating that the vessels in RP tumors lacked pericyte coverage (Figure 2E). Second, tumor cells in RP mice exhibited typical NE-SCLC morphology: small size, high nuclear/plasma ratio, and dense nuclei. In contrast, tumor cells in RPPa mice exhibited a morphology resembling non-NE carcinomas: larger size, containing 1 or more prominent nucleoli with paranucleolar chromatin clearing (Figure 2F). Third, tumor cells in RP mice expressed ASCL1, NCAM1, and SYP, whereas tumor cells in RPPa mice lacked these proteins but expressed KRT7 and YAP (Figure 2G). Fourth, the RPPa tumors exhibited reduced drug sensitivity to a combination of cisplatin and etoposide, the standard chemotherapy for SCLC, compared with the RP tumors as measured by cleaved caspase-3 and phosphorylated H2AX levels (Supplemental Figure 3, A–C). Fifth, the RPPa tumors exhibited increased immune infiltration compared with the RP tumors (Supplemental Figure 3D). It has been previously reported that increase in non-NE cells is associated with more immune infiltration (36, 37). These collective data suggest that PAX5 deletion may not affect tumor initiation but caused loss of NE features during progression of *Rb1/Trp53* deficient tumors.

We next examined the effect of chemotherapy on RP tumors. Immunohistochemical analysis showed reduced PAX5 expression in RP tumors 2 weeks after treatment with etoposide/cisplatin (Supplemental Figure 3E), suggesting that chemotherapy may repress PAX5 expression. Concurrently, increased expression of HES1 and YAP, along with decreased expression of ASCL1 and NCAM1, were observed (Supplemental Figure 3E).

### Overexpression of PAX5 partially confers lung adenocarcinomas with SCLC phenotype.

We next explored the role of PAX5 in transformation from adenocarcinoma to SCLC. PAX5 expression was associated with impaired survival in lung adenocarcinomas in Kaplan-Meier Plotter database (Supplemental Figure 4A). Interrogation of a published transcriptome dataset of biopsies of resistant lung adenocarcinoma that transformed to SCLC or that maintained adenocarcinoma histology (14) revealed that *PAX5* expression increased and preceded induction of NE transcription factors (*NEUROD1*, *POU3F2*, *INSM1*, *HES6*) during SCLC transition (Supplemental Figure 4B).

We then generated TetO-*hPAX5* knockin mice and crossed them with CC10-rtTA TetO-*hEGFR*^ex19del/T790M^ (EC) mice to obtain triple-transgenic animals expressing both mutant human *EGFR* (a driver of lung adenocarcinoma) and human *PAX5* (EPC) in a lung-specific and inducible manner. Upon doxycycline administration, transgene expression is induced specifically in developing and mature respiratory epithelium (38, 39). H&E staining of entire lung tissue sections from 3 EC and 3 EPC mice 1 month after doxycycline administration revealed that the lungs of EPC mice developed numerous tumor nodules, whereas only a single nodule was observed in the lungs of EC mice (Supplemental Figure 4C). Additionally, overexpression of PAX5 enhanced clonogenicity of human bronchial epithelial cells (BEAS-2B and 16HBE) and EGFR-mutant lung cancer cells (PC-9) in soft agar (Supplemental Figure 4, D–F). These data suggest that PAX5 promotes more aggressive behavior in lung carcinomas. Further analysis of The Cancer Genome Atlas database revealed no correlation between *PAX5* expression and *EGFR* mutation status (Supplemental Figure 5A), indicating that PAX5 may function in a broader context.

**Figure 3:**
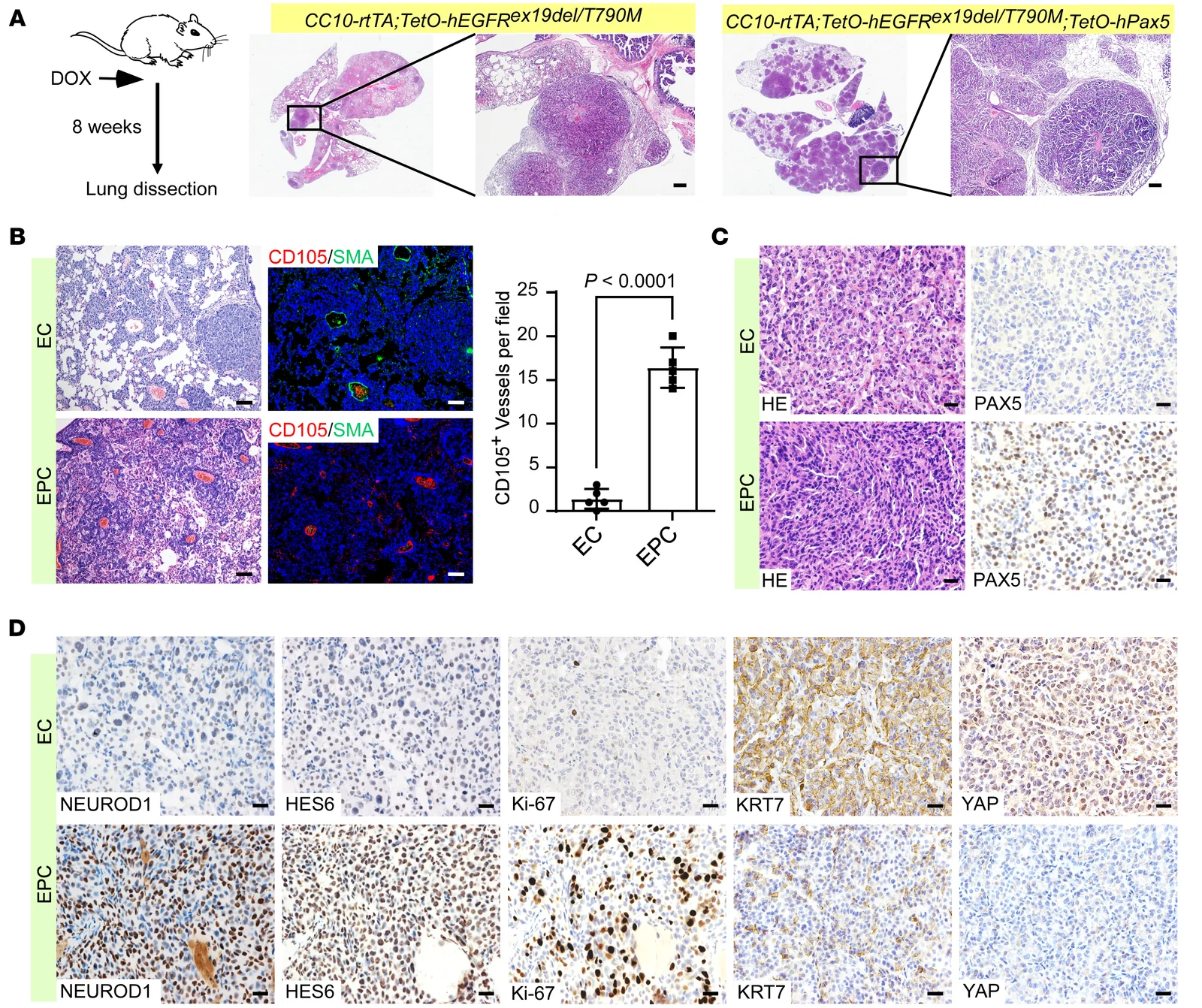
PAX5 transforms lung adenocarcinoma into SCLC. (**A**) Representative H&E staining and scanning images of lung tumor nodules developed in CC10-rtTA TetO*-hEGFR*^ex19del/T790M^ (EC) bitransgenic and CC10-rtTA TetO-*hEGFR*^ex19del/T790M^ TetO-*hPAX5* (EPC) tritransgenic mice at 8 weeks after doxycycline treatment (*n* = 5 independent mice per group). Scale bar, 200 μm. (**B**) Representative H&E staining and immunofluorescence images of CD105 and α-SMA in tumor sections from EC and EPC mice (left panels, *n* = 5 independent mice per group), with the corresponding quantification of CD105^+^α-SMA^–^ vessels (right panel). Scale bar, 100 μm. Data are shown as mean ± SD (*n* = 5 mice per group); unpaired, 2-tailed Student’s *t* test. (**C** and **D**) Images of H&E staining and immunostaining of tumors developed in EC and EPC mice. Scale bar, 20 μm.

After 2 months of doxycycline administration, EC mice developed tumors, and EC tumors and EPC tumors exhibited substantial histological differences. Compared with EC tumors, which showed a peripheral adenocarcinoma phenotype, EPC tumors exhibited rapid tumor growth (Figure 3A), increased abnormal vessels (Figure 3B), SCLC-like histopathology (smaller size, oat shape, and dense nuclei), reduced KRT7 and YAP expression, increased expression of NEUROD1 and HES6 (expression of ASCL1, NCAM1, and SYP was not detected) (Figure 3, C and D), and increased sensitivity to cisplatin/etoposide treatment (Supplemental Figure 5B). These results suggest that expression of PAX5 in *EGFR*-mutated lung adenocarcinoma partially endows adenocarcinoma with NE features. PAX5 was not induced in EC tumors 2 weeks after erlotinib treatment, as assessed by immunohistochemical analysis (Supplemental Figure 5C). This indicates that erlotinib may not directly induce PAX5 expression, which is consistent with the clinical observation that transformation of *EGFR*-mutant NSCLCs into SCLCs following EGFR inhibition occurs in only a minority of patients (13).

RP tumors expressed ASCL1, whereas EPC tumors expressed NEUROD1. To understand whether PAX5 specifically regulates ASCL1- or NEUROD1-type SCLC, we assessed the correlation between *PAX5* with these 2 markers in our scRNA-seq data and established different subtypes of SCLC lines. No correlation was observed (Supplemental Figure 5D), suggesting that this differential expression pattern may arise from distinct cells of origin or distinct oncogenic contexts in different mouse models.

### PAX5-induced angiogenic microenvironment contributes to the maintenance of NE identity.

**Figure 4:**
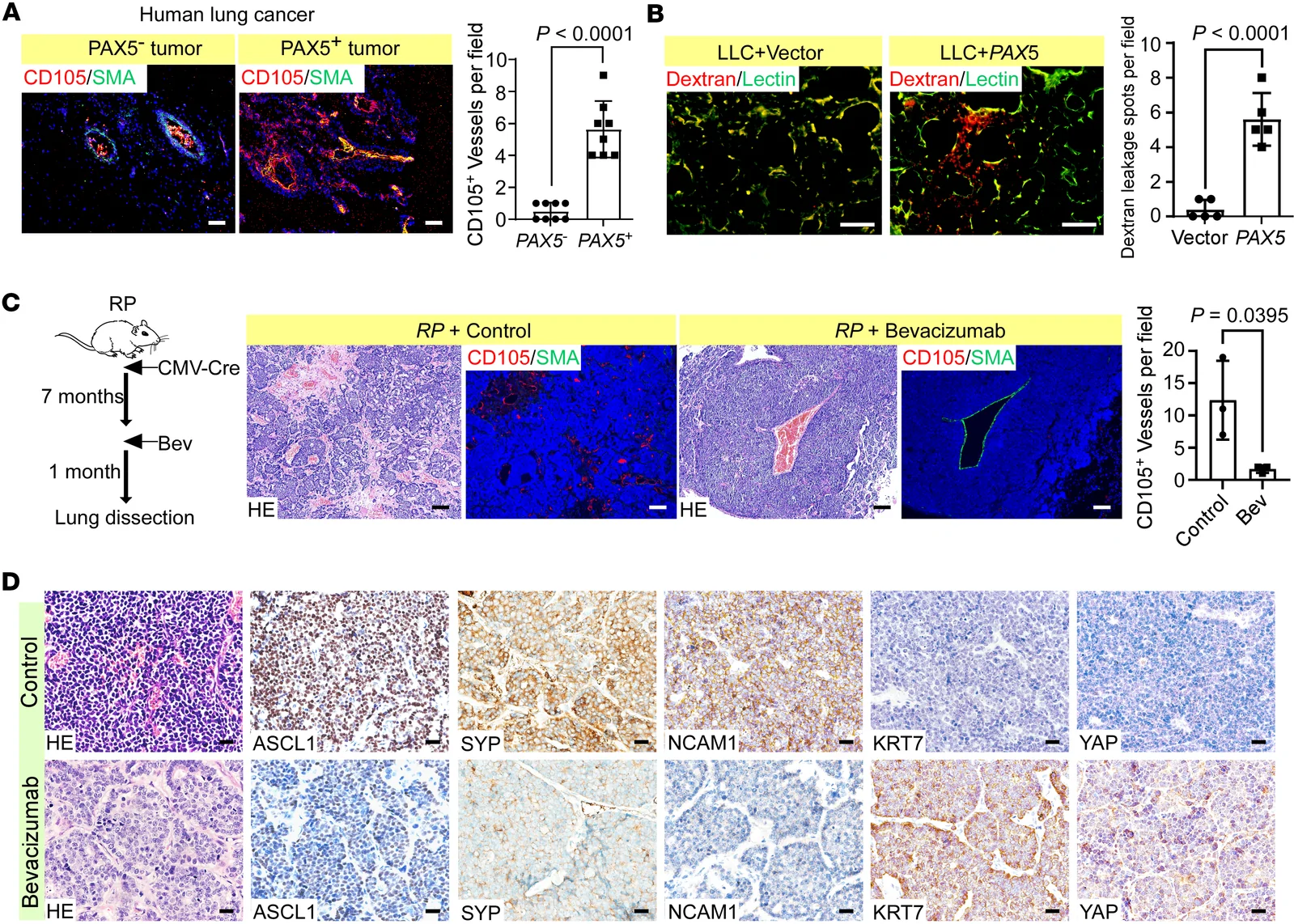
Antiangiogenic drug bevacizumab blocks PAX5-induced SCLC transformation. (**A**) Representative immunostaining images of CD105 and α-SMA in human primary lung cancer tissues stratified by PAX5 expression (left panels, *n* = 8 independent patients per group), with the corresponding quantification of CD105-positive vessels (right panel). Scale bar, 100 μm. Data are shown as mean ± SD (*n* = 8 patients per group); unpaired, 2-tailed Student’s *t* test. (**B**) Mice carrying control or *PAX5*-expressing tumors were intravenously injected by lectin-FITC followed by dextran-rhodamine. Representative images showing dextran leakage from vessels (left panels, *n* = 5 independent mice per group), with the corresponding quantification of vessels (right panel). Scale bar, 100 μm. Data are shown as mean ± SD (*n* = 5 mice per group); unpaired, 2-tailed Student’s *t* test. (**C**) Representative H&E staining and immunofluorescence images of CD105 and α-SMA in tissue sections of tumors developed in RP mice treated with saline or bevacizumab (Bev) (left panels, *n* = 3 independent mice per group), with the corresponding quantification of vessels (right panel). Scale bar, 100 μm. Data are shown as mean ± SD (*n* = 3 mice per group); unpaired, 2-tailed Student’s *t* test. (**D**) Images of H&E staining and immunostaining of tumors developed in RP mice treated with saline or bevacizumab. Scale bar, 20 μm.

Notably, PAX5 exhibited a proangiogenic role in both RP and EPC genetically engineered mouse models. Furthermore, the CD105-positive vessels in RP tumors were also CD31 positive, evaluated by immunohistological analysis in serial sections of tumors (Figure 2E and Supplemental Figure 6A), indicating that these CD105-positive vessels are normal vessels instead of vasculogenic mimicry. To validate the role of PAX5 in the angiogenic microenvironment, we deleted *PAX5* in H1155 cells and overexpressed *PAX5* in A549 cells, then subcutaneously inoculated the cancer cells into BALB/c nude or NOD/SCID mice. Consistently, significantly more vessels were observed in *PAX5*-expressing A549 xenografts and in *PAX5*-expressing H1155 xenografts than in their corresponding controls (Supplemental Figure 6, B and C). Likewise, significantly more vessels were observed in *PAX5*-expressing LLC allografts. Again, these vessels were positive for CD105 but negative for α-SMA (Supplemental Figure 6D). Consistent with the findings in xenograft and allograft models and genetically engineered models, more CD105^+^α-SMA^–^ vessels were found in PAX5-positive human primary lung cancer tissues than in those that were PAX5 negative (Figure 4A). Therefore, in human lung cancers and 5 mouse models, PAX5 induces an intense angiogenic response. In keeping with the defect in mural cell coverage, we also found dextran leakage in lungs from mice harboring *PAX5*-expressing LLC tumors but not in lungs from mice harboring control tumors (Figure 4B). Thus, PAX5 increases vascular density and permeability, characteristics associated with tumor microvessels.

Vascular abnormalities within tumors lead to a microenvironment characterized by interstitial hypertension, hypoxia, and acidosis, which can alter the intrinsic characteristics of tumor cells (40). Consistently, immunohistochemical analysis showed increased expression of HIF-1α, a TF that is activated in response to hypoxia, in PAX5-expressing RP and EPC tumors (Supplemental Figure 6, E and F). Moreover, hypoxia treatment upregulated expression of a series of NE genes including *SYP*, *NCAM1*, *CHGA*, *INSM1*, *ASCL1*, and *SYN1* in PAX5-expressing A549 and PC-9 cells (Supplemental Figure 6, G–I), suggesting that hypoxia contributes to this PAX5-induced NE lineage transition.

To investigate whether the PAX5-induced aberrant vasculature plays a role in determining NE-SCLC identification in vivo, we treated tumor-bearing RP mice with bevacizumab, a blocking monoclonal antibody against VEGFA, to normalize the tumor vasculature. As expected, 1 month’s treatment with bevacizumab reduced CD105^+^α-SMA^–^ tumor vessels (Figure 4C). Importantly, RP tumors harvested from bevacizumab-treated RP mice exhibited decondensed nuclei; decreased expression of ASCL1, NCAM1, and SYP; and increased expression of KRT7 and YAP (Figure 4D), indicating that bevacizumab treatment reduced NE features of SCLCs. Although bevacizumab can block the lineage transition, neither NE genes were upregulated nor non-NE genes were downregulated upon VEGF treatment in A549 cells (Supplemental Figure 6J). Thus, an angiogenic microenvironment is important to maintain NE phenotype of SCLCs.

### PAX5 induces an angiogenic secretome.

**Figure 5:**
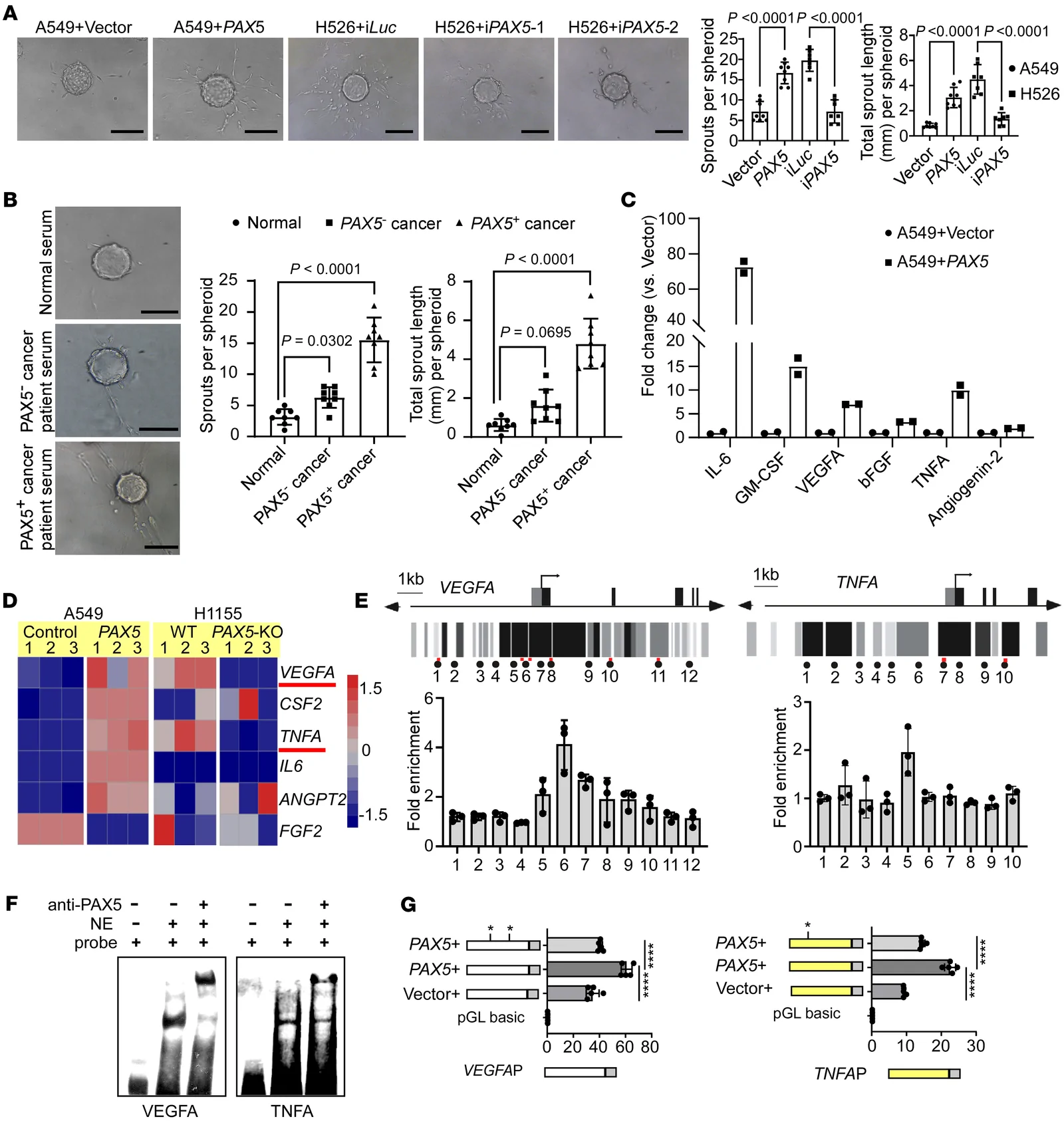
PAX5 upregulates proangiogenic gene expression. (**A**) CM from A549-*PAX5* or H526-sh*PAX5* cells was added into HMVEC spheroids. Representative images of A549 (*n* = 8 per group) and H526 (*n* = 7 per group) spheroids (left panels) and the corresponding quantification (right panels). Scale bar, 200 μm. Data are mean ± SD; unpaired, 2-tailed Student’s *t* test. (**B**) Sera from patients with varying PAX5 levels were added into HMVEC spheroids. Representative images (left panels, *n* = 8 per group) and the corresponding quantification (right panels). Scale bar, 200 μm. Data are mean ± SD; 1-way ANOVA followed by Dunnett’s multiple comparisons test (vs. normal group). (**C**) Protein array analysis of proangiogenic factors secreted by control and *PAX5*-expressing A549 cells. All factors were assayed in duplicate, representative of 2 independent experiments. (**D**) Heatmap shows transcription of indicated proangiogenic factors. (**E**) ChIP analysis of PAX5 binding to *VEGFA* and *TNFA* loci. DNase I–hypersensitive sites (ENCODE, vertical lines) and PAX5-binding consensus sites (red squares) in the ±5 kb region of the transcriptional start site were analyzed. ChIP-qPCR was performed using FLAG antibody in A549 cells transfected with *PAX5*-FLAG. Data are mean ± SD of 3 independent experiments, each performed in duplicate. (**F**) EMSA shows mobility shift of the probe and a supershift following anti-PAX5 treatment. Representative of 2 independent experiments. (**G**) Luciferase reporter activity with promoters of indicated genes cotransfected with vector or *PAX5*. Stars indicate mutation of *PAX5* binding consensus site. A representative from 3 independent experiments is shown. Data are mean ± SD of 5 technical replicates; 1-way ANOVA followed by Dunnett’s multiple comparisons test (vs. PAX5 group). ****P < 0.0001.

To further confirm the proangiogenic role of PAX5 and understand the underlying mechanism, we collected conditioned medium (CM) from cancer cells and the serum from patients with lung cancer (Supplemental Table 2) and performed a 3D vascular sprouting assay using primary human microvascular endothelial cells (HMVECs). We found that the CM collected from the PAX5-expressing cancer cells (SCLC cells and adenocarcinoma cells transduced with *PAX5*) induced more sprouting than the CM collected from the PAX5-negative cancer cells (adenocarcinoma cells and SCLC cells transduced with *PAX5* shRNA) (Figure 5A and Supplemental Figure 7A). In addition, serum from patients with PAX5-positive lung cancer consistently induced more sprouting than that from the patients with PAX5-negative lung cancer (Figure 5B). Together, these results indicate that PAX5 may induce vascularization by promoting the secretion of proangiogenic factors.

Given that antiangiogenic treatment specific to VEGFA had a marked effect on NE differentiation in RP tumors, we tested whether PAX5 induced a broad angiogenic secretome beyond VEGFA. We conducted a protein array analysis of the CM collected from cancer cells transduced with *PAX5*, and we identified a subset of factors including IL-6, GM-CSF, VEGFA, bFGF, TNFA, and angiopoietin-2 (Figure 5C and Supplemental Figure 7B). Except for *FGF2*, all the other PAX5-induced proangiogenic factors were upregulated transcriptionally. Among those, *VEGFA* and *TNFA* were transcriptionally downregulated in H1155 cells upon *PAX5* deletion (Figure 5D). PAX5 bound directly to the promoter regions of these 2 genes, as evaluated by chromatin immunoprecipitation (ChIP) and EMSA, and enhanced the association of H3K4me3 and H3K9ac, 2 histone modifications that tightly correlate with active gene transcription, with *VEGFA* and *TNFA* (Figure 5, E and F, and Supplemental Figure 7C). Mutations of the PAX5 binding sites in the promoter regions of these genes blocked PAX5-dependent promoter activity in luciferase assays (Figure 5G). Thus, PAX5 upregulates proangiogenic genes and induces aberrant vascularization of the tumor microenvironment.

### Transcriptional landscape is regulated by PAX5.

**Figure 6:**
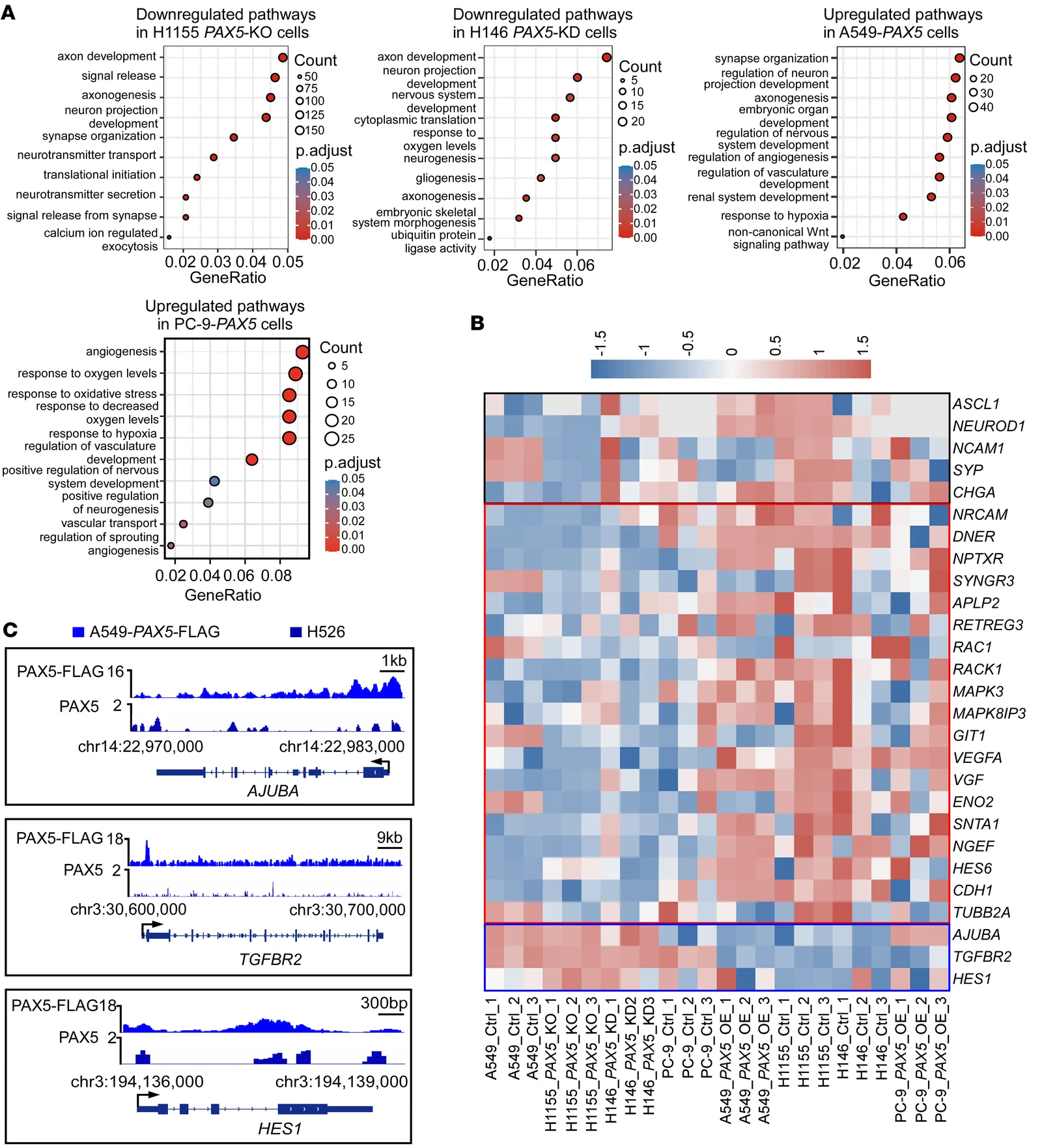
Transcriptional landscape regulated by PAX5. (**A**) GO analysis showing PAX5-associated signaling pathways in H1155, H146, A549, and PC-9 cells. (**B**) Heatmap showing transcription of indicated genes. (**C**) ChIP-seq showing association of PAX5 in the indicated genes. Two independent experiments were performed, and data from 1 representative experiment are shown.

To explore the effect of PAX5 on established cancer cells, we expressed *PAX5* in A549 and PC-9 cells and knockout/knockdown *PAX5* in H1155 and H146 cells (Supplemental Figure 8A) and analyzed the resultant transcriptional profile using RNA-seq. Gene ontology (GO) analysis revealed that the top downregulated signaling pathways upon *PAX5* knockout/knockdown in H1155 and H146 cells were related to axon development, synapse organization, neural system development, and neurotransmitter secretion (Figure 6A). Consistently, signaling pathways of neural system development were upregulated upon *PAX5* overexpression in A549 and PC-9 cells (Figure 6A). In addition, genes related to angiogenesis and response to hypoxia were also upregulated upon *PAX5* overexpression. Specifically, *NRCAM*, *HES6*, *VGF*, *SNTA1*, *CDH1*, and *TUBB2A* genes were upregulated by *PAX5*. Notably, non-NE genes including *AJUBA*, *TGFBR2*, and *HES1* were downregulated by *PAX5* (Figure 6B). ChIP-seq analysis using FLAG antibody in A549-*PAX5* cells and PAX5 antibody in H526 cells demonstrated the occupancy of PAX5 at the promoters of these non-NE genes (Figure 6C). Expression of *ASCL1*, *NEUROD1*, *SYP*, and *CHGA* was not associated with *PAX5* (Figure 6B), suggesting that these genes were not directly regulated by *PAX5*. These data indicate that besides activating the NE program via creating an angiogenic microenvironment, PAX5 can regulate neural genes’ expression and repress several non-NE genes in cancer cells. In addition, we observed that PAX5 positively regulated its own expression, because PAX5 overexpression in A549 cells increased the enrichment of H3K27ac (a histone mark associated with active transcription) at the endogenous *PAX5* locus (Supplemental Figure 8B). This self-activation mechanism explains why PAX5 expression persists in EPC tumors despite CC10 promoter repression (Supplemental Figure 8C).

### PAX5 promotes NE transformation in prostate carcinoma.

**Figure 7:**
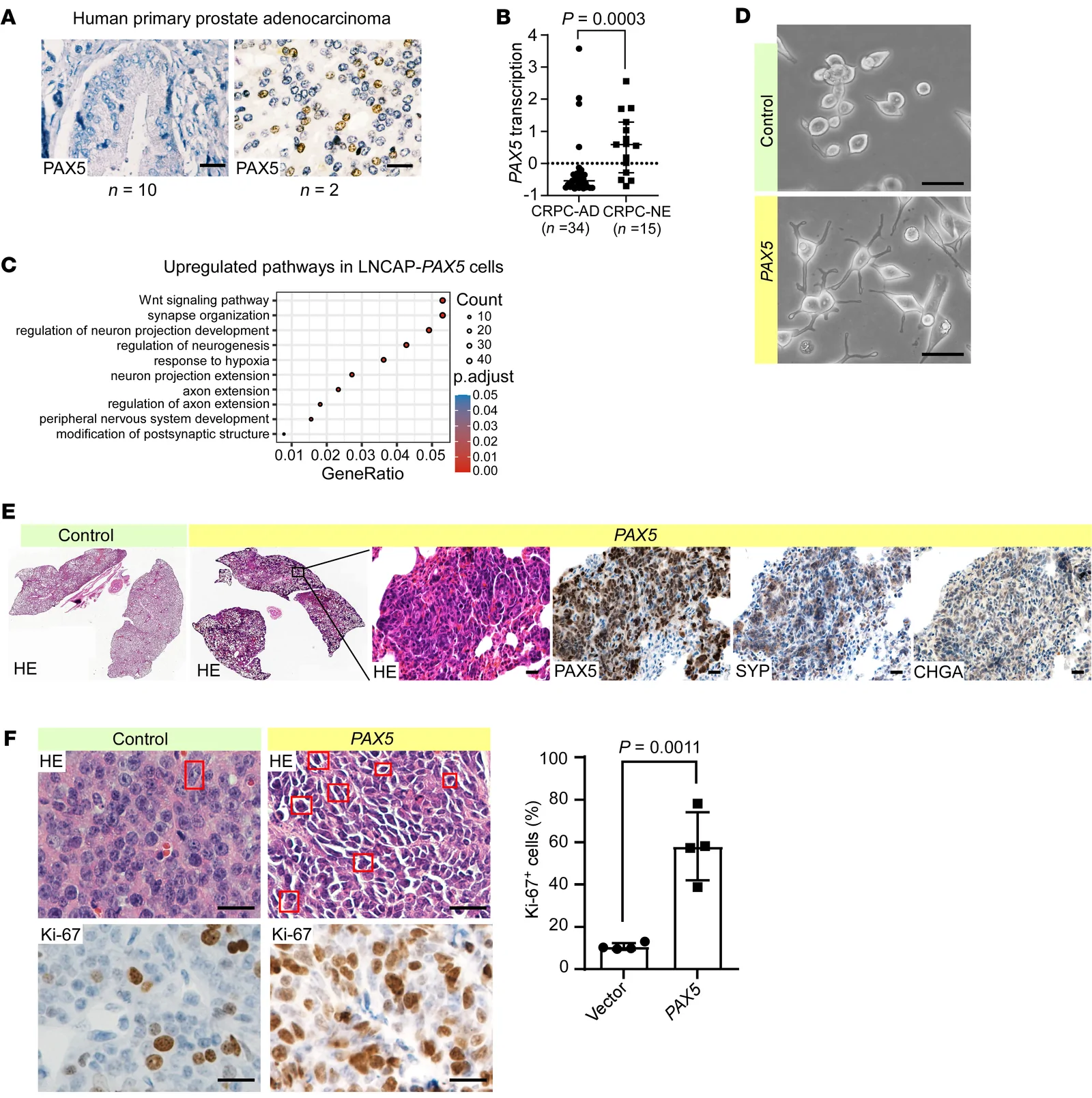
PAX5 promotes the transformation of prostate adenocarcinoma to SCPC. (**A**) Representative images of immunostaining with anti-PAX5 antibody in human primary prostate adenocarcinomas. Scale bar, 20 μm. (**B**) Quantitation of *PAX5* expression in CRPCs that retained adenocarcinoma phenotype and CRPCs that transformed into SCPC generated by Beltran et al. (15). Data are presented as median with interquartile range; 2-tailed Mann-Whitney test. (**C**) GO analysis of *PAX5*-associated signaling pathways in LNCAP cells. (**D**) Morphology of control and PAX5-expressing LNCAP cells. Scale bar, 50 μm. (**E** and **F**) Control or PAX5-expressing RM-1 cells were injected into the prostate of C57BL/6 mice. Lungs and primary tumors were analyzed at 2 weeks. H&E and immunostaining for PAX5, SYP, and CHGA of lung tissue sections (**E**) and H&E and immunostaining for Ki-67 of primary tumors (**F**). Scale bar, 20 μm. Cancer cells in red boxes are mitotic. Ki-67–positive cells were quantified. Data are shown as mean ± SD (*n* = 4 independent mice per group); unpaired, 2-tailed Student’s *t* test.

SCLC and SCPC have been reported to share transcriptional and morphologic similarities (41). We therefore explored whether PAX5 plays a similar role in SCPC transdifferentiation. Of 12 human primary prostate adenocarcinomas (PrADs), we identified 2 tumors that expressed PAX5 (Figure 7A). PAX5 staining was heterogeneous with a scattering of individual PAX5-positive cells. Transcriptome analysis of castration-resistant prostate cancer (CRPC) samples (including 34 CRPCs that maintained the adenocarcinoma phenotype and 15 CRPCs undergoing NE transformation) generated by Beltran et al. (15) revealed that tumors undergoing NE conversion expressed much higher levels of *PAX5* than those that maintained the adenocarcinoma phenotype (Figure 7B). GSEA showed that the top upregulated signaling pathways associated with *PAX5* expression were related to cell cycling (Supplemental Figure 9A), consistent with observations in lung cancers. We then overexpressed PAX5 in LNCAP human PrAD cells that lack endogenous *PAX5* expression and analyzed the resulting transcriptome. Similar to its effect on lung adenocarcinoma cells, *PAX5* expression led to the activation of gene sets related to synapse organization, axon extension, and neuron projection development in LNCAP cells (Figure 7C). Consistent with this result, PAX5 induced an SCNC morphology of LNCAP cells (Figure 7D).

We next examined the function of PAX5 in prostate cancer using orthotopic tumor implantation. RM-1 mouse prostate carcinoma cells were lentivirally transduced with *PAX5*, mixed with mouse prostate cancer-associated fibroblasts, and transplanted into the prostates of C57BL/6 mice. Two weeks after injection, all 4 mice bearing *PAX5* tumors had lung metastases, whereas mice carrying the control tumors did not (Figure 7E). Notably, the lung metastases demonstrated histological features of SCPC and uniform expression of NE markers (Figure 7E), suggesting that PAX5 may promote metastasis by conferring NE phenotype. In addition, morphological differences were observed between control and *PAX5* tumors at the primary site. Specifically, while control tumors exhibited histological features of adenocarcinoma, PAX5-expressing tumors showed characteristics of SCPC: an oat shape, scant cytoplasm, and increased numbers of mitotic cells as confirmed by Ki-67 immunostaining (Figure 7F). Thus, as a key regulator of both neural system and lymphocyte development, PAX5 transforms adenocarcinoma into SCNC independent of the tissue of origin.

**Figure 8:**
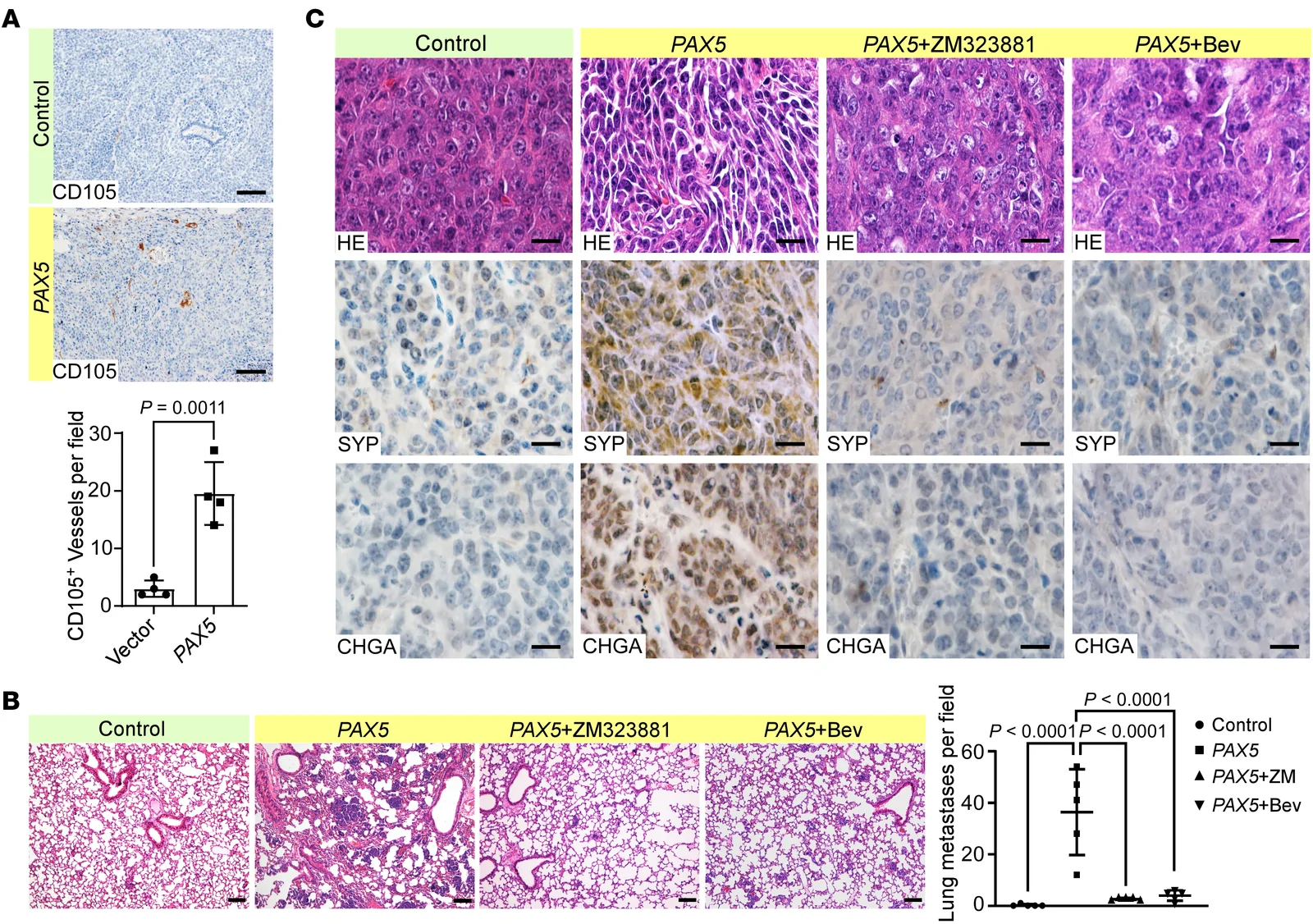
PAX5 drives tumor angiogenesis and promotes metastasis in SCPC transformation. (**A**) Representative CD105 immunostaining images in control and *PAX5* primary tumors (top panels, *n* = 4 independent mice per group) with the corresponding quantification of CD105^+^ vessels (bottom panel). Scale bar, 100 μm. Data are shown as mean ± SD (*n* = 4 mice per group); unpaired, 2-tailed Student’s *t* test. (**B**) Representative H&E staining of lung tissue sections showing metastases with or without antiangiogenic drugs (left panels, *n* = 5 independent mice per group) and the corresponding quantification (right panel). Scale bar, 100 μm. Data are shown as mean ± SD (*n* = 5 mice per group); 1-way ANOVA followed by Dunnett’s multiple comparisons test (vs. *PAX5* group). (**C**) H&E staining and immunostaining for NE markers in RM-1 tumors with or without antiangiogenic drugs. Scale bar, 20 μm.

As seen in lung carcinomas, PAX5-expressing prostate tumors also contained more CD105-positive vessels at the primary site than the control tumors (Figure 8A). The correlation analysis using Beltran et al.’s data (15) showed the positive correlation between *PAX5* expression and angiogenic factors *TNFA* (*r* = 0.534) and *TGFB1* (*r* = 0.378) (Supplemental Figure 9B). Bevacizumab and another antiangiogenic drug, ZM323881 HCl (an inhibitor of VEGFR), blocked PAX5-induced RM-1 tumor metastasis and SCPC transformation (Figure 8, B and C), confirming a critical role for active angiogenesis in PAX5-induced SCPC as well as SCLC transformation.

## Discussion

Solid tumors gain aggressive characteristics such as metastasis and drug resistance, in part by acquiring lineage features normally restricted to other cell types. In this study, we generated scRNA-seq data of a primary untreated SCLC and identified 2 NE clusters (*ASCL1*-positive and *NEUROD1*-positive clusters) and a non-NE cluster. These different subtypes of SCLCs exhibit similar CNV patterns, suggesting that they are derived from a single clone and that lineage transition can occur between different subtypes. Starting with these scRNA-seq data, we identified TF PAX5 as an important factor contributing to the lineage transition between NE and non-NE cancer cells, consistent with a previous study that also identified PAX5 as an important TF to induce an NE phenotype in prostate cancers by comparing chromatin accessibility, epigenetic signatures, and gene expression between prostate adenocarcinoma and NE-like prostate cancers (42).

Four subtypes of SCLCs have been classified based on their differential expression of *ASCL1*, *NEUROD1*, and *POU2F3* or low expression of all 3 TFs (6, 18–21). Our single-cell transcriptomic map confirmed this heterogeneity and further demonstrated the coexistence of different subtypes within primary human SCLC tumors. The expression of PAX5 in SCLCs has been previously observed (43–47). Our single-cell analysis revealed that *PAX5* is predominantly expressed in NE-SCLC cells, although its expression level is relatively low compared with that of *ASCL1* and *NEUROD1*, but not in non–NE-SCLC cells. Further, our mechanistic studies demonstrated a causative role of PAX5 in preserving NE status.

PAX5 was not able to activate NE genes in established lung cancer cell lines, suggesting that other TFs are involved and that microenvironment plays important role in this PAX5-mediated NE differentiation. Although it has long been noted that active angiogenesis is associated with SCNC (34, 48), what caused the active angiogenesis in SCNCs and the role of active angiogenesis in SCNC plasticity is a longstanding question. Our study demonstrates that the expression of PAX5 in malignant cells promotes angiogenic secretome and generates many abnormal vessels that lack pericyte coverage, contributing to the angiogenic microenvironment. Importantly, blocking angiogenesis can reduce NE features in an RP SCLC mouse model and prevent NE transformation in a prostate xenograft model, suggesting the important role of an angiogenic environment in SCNC lineage transition. This finding provides a mechanistic explanation for the limited efficacy observed in recent clinical trials combining angiogenesis inhibitors with chemotherapy for patients with SCLC (49–52). Furthermore, this finding suggests that adding angiogenesis inhibitors to standard chemotherapy may benefit patients with adenocarcinoma by preventing lineage transition from adenocarcinoma to NE-SCLCs, highlighting its translational implications. How active angiogenesis regulates lineage transition is not currently solved in this study. We found that HIF-1α was markedly upregulated in RP and EPC tumors compared with RPPa and EC tumors and that hypoxia increased expression of multiple NE genes in PAX5-expressing lung cancer cells. It has been reported that HIF-1α is expressed in NE prostate tumors (53) and upregulates the expression of NE genes such as Hes6, Sox9, and Jmjd (54). FGF9, which can be upregulated at the translational level by hypoxia (55), contributes to NE lineage transdifferentiation (56). ADORA2A, a G protein–coupled receptor that can be upregulated by HIF-2α (57) or NF-κB (58), is also involved in SCLC transformation (59).

PAX5 is normally expressed in the mesencephalon and spinal cord in the developing central nervous system during embryogenesis (24). In adult life, PAX5 expression is only detected in B lymphocytes and at early but not late stages of B cell differentiation (60, 61). Germline mutation of *Pax5* in mice impairs morphogenesis of the posterior midbrain and completely blocks B cell development at an early precursor stage (25, 26), demonstrating its critical role in determining neuronal and B cell lineage. Here, we show that PAX5 recapitulates these developmental actions in malignant states, explaining the propensity of SCNCs to exhibit multiple unique differentiation features compared with other epithelial cancers. First, SCNC cells express an NE program, which is important for both initiation and progression of SCNC tumors. We now demonstrate that this program can be directly or indirectly activated by PAX5. On the one hand, PAX5 directly regulates expression of a series of neuronal genes in cancer cells. On the other hand, PAX5 generates an angiogenic microenvironment that can induce NE transcriptional program. Second, SCNCs also share histological and clinical similarities with aggressive lymphomas, including metastatic ability and interaction with angiogenic vasculature. Accordingly, SCLC had been classified as lymphoma until 1926 (4, 62), and even now clinical pathologists risk misdiagnosis of SCLC as lymphoma (63). Third, SCNCs share some drug sensitivities with lymphoma, such as the histone deacetylation inhibitor vorinostat that is effective for both SCNCs and lymphoma (64, 65). Finally, SCNCs also display many phenotypic characteristics of lymphoma cells when cultured in vitro, such as small size, anchorage independence, and a propensity to grow in suspension (28). In general, our finding that PAX5 is a critical determinant of NE differentiation in SCNC tumors provides evidence that cell type–specific TFs may play a major role in driving lineage plasticity during the epigenetic evolution of individual human cancer cells.

Paradoxically, PAX5 acts as a tumor suppressor in B-lineage acute lymphoblastic leukemia (B-ALL), in which it is one of the most mutated genes (66, 67). Loss-of-function mutations of this gene cause downregulation of B lineage–specific genes, impairing B cell differentiation and promoting transformation to a leukemic state (68–73). Similar loss of function or hypomorphic mutations of other genes required for B cell maturation such as *EBF1* and *IKZF1* also lead to B-ALL (66), suggesting that PAX5 functions as a tumor suppressor by advancing B cell differentiation beyond a highly proliferative progenitor stage. In the context of epithelial carcinomas, however, PAX5 lacks normal developmental function, and its presence would not be expected to gainsay mutational defects in cell cycle control. Notably, PAX5 confers chemosensitivity in SCLC but therapeutic resistance in *EGFR*-mutant lung adenocarcinoma (LUAD). PAX5 expression induces an NE status in both SCLC and LUAD. In SCLCs, NE status confers chemosensitivity; however, in *EGFR*-mutant LUAD, it leads to resistance to EGFR inhibitors. Therefore, the effect of PAX5 on cancer cell responses to chemotherapy is likely indirect and primarily mediated through its role in promoting NE lineage transition.

## Methods

### Sex as a biological variable.

Lung cancer studies included both sexes in primary mouse models and human samples, but sex was not formally analyzed as a biological variable in these experiments. Subcutaneous tumor implantation was performed in female mice to minimize handling-induced stress and variability during serial measurements, and these findings are expected to also be applicable to male mice. In contrast, orthotopic prostate tumor experiments were conducted in male mice, consistent with the male-specific anatomy of the prostate, and human prostate cancer specimens were obtained exclusively from male patients. Therefore, the findings from the prostate studies are not expected to be relevant for women.

### Reagents and tools.

Reagents, cell lines, and software used in this study are detailed in Supplemental Table 3. Further information can be found in Supplemental Methods.

### Human samples.

Human lung cancer tissues were collected at Tianjin Medical University Cancer Hospital. Human prostate cancer tissues were collected at Tianjin Medical University Second Hospital.

### Single cell processing.

One SCLC tissue specimen was collected at Tianjin Medical University Cancer Hospital. The resected tumor was transported in MACS Tissue Storage Solution on ice immediately after surgical procurement. A small fragment was fixed in 4% paraformaldehyde at 4°C overnight and then paraffin-embedded for immunohistochemical analysis. The remaining tumor was minced into tiny cubes (<1 mm^3^), then enzymatically digested with MACS tumor dissociation kit for 60 min on a rotor at 37°C, according to the manufacturer’s instructions. After digestion, the cells were centrifuged at 400 × *g* at 4°C for 5 min, then resuspended in 100 U/mL DNase I and incubated at 37°C for 15 min. After filtering and centrifugation at 300 × *g* at 4°C for 5 min, the pellet was suspended in red blood cell lysis buffer and incubated on ice 2 min to lyse red blood cells. Finally, the cells were spun down and resuspended in culture medium (DMEM/F12 supplemented with 10% FBS).

Isolated cells were stained with CD45-PE, CD31-PE, and DAPI according to manufacturer recommendations. Cells were washed with PBS with 2% FBS, and DAPI, CD45, and CD31 triple-negative cells were sorted on a BD FACSAria II for further analysis.

For droplet-based scRNA-seq, single cells were captured and barcoded in a 10x Genomics Chromium Controller. scRNA-seq libraries were prepared using Chromium Single Cell 3′v2 Reagent Kit. Sequencing libraries were loaded on an Illumina NovaSeq with 2 × 150 paired-end kits. The FASTQ files were analyzed with the Cell Ranger Software Suite against the GRCh38 human reference genome.

To visualize the processed data obtained from Cell Ranger analysis, cell clustering was performed using the Seurat suite (version 3.1). The filtered gene-barcode matrix generated by Cell Ranger count for the sample was imported into Seurat. Low-quality or dying cells were excluded by retaining only cells with adequate molecular read counts and gene detection (nCount_RNA < 50,000; nFeature_RNA > 200 and < 9,000), along with a low proportion of mitochondrial transcripts (percent.mt < 25%). The data were then normalized and scaled, followed by linear dimensionality reduction via principal component analysis. Subsequently, cell clustering was conducted and results were visualized using UMAP.

### Binding motif enrichment analysis.

In the SCLC scRNA-seq dataset, the FindMarkers function from the Seurat package was utilized to identify the log fold-changes (logFCs) of all genes across the entire expression profile between the NE-SCLC (cluster 0, 1, 2, 3, 4) and non–NE-SCLC (cluster 5) groups. The resulting genes were then ranked based on their logFC values and used as input for the GSEA function from the clusterProfiler package. GSEA was performed using the C3: TFT gene set (comprising all TF target prediction gene sets, which represents a combined superset of both GTRD prediction methods and legacy sets) to compare the 2 groups. Subsequent visualization of the enrichment results was carried out using the gseaplot2 function from the enrichplot package.

### Inferred CNV analysis.

UMAP analysis revealed 12 transcriptionally distinct clusters, including cancer-associated fibroblasts, myoblasts, normal epithelial cells, and lung cancer cells. To distinguish malignant cells from nonmalignant epithelial cells, we first excluded the CAFs and myoblasts, which show distinct transcription profiles from epithelial cells, and then inferred CNVs from expression profiles of the remaining single cells by averaging expression over stretches of 100 genes on their respective chromosomes. As a control, we included scRNA-seq profiles of epithelial cells from normal human lung (74). In the CNV hierarchical cluster analysis, the normal lung epithelial cells clustered with single cells of clusters 6, 7, 8, and 9, which were classified as club cells, ciliated cells, alveolar type 1 cells, and alveolar type 2 cells according to the expression of specific cell markers. Clusters 0, 1, 2, 3, 4, and 5 were classified as malignant cells.

### Generation of Rb1^fl/fl^ Trp53^fl/fl^ Pax5^fl/fl^ mice.

We crossed *Rb1^fl/fl^ Trp53^fl/fl^* (RP) to *Pax5^fl/fl^* mice to generate *Rb1^fl/fl^ Trp53^fl/fl^ Pax5^fl/fl^* (RPPa). *Rb1^fl/fl^* *Trp53^fl/fl^* mice were provided by Hongbin Ji (Institute of Biochemistry and Cell Biology, Chinese Academy of Sciences, Shanghai, China). Primers for genotyping are listed in Supplemental Table 4. At 6~8 weeks of age, RP and RPPa mice were fully anesthetized and infected with 2.5 × 10^7^ PFU of adenovirus-CMV-Cre recombinase by intratracheal delivery.

### Generation of TetO-hPAX5 mice.

To generate the mice with inducible expression of human *PAX5* (*hPAX5*) by doxycycline, we performed site-specific gene integration via CRISPR/Cas9 system. The genomic locus of choice for targeted knockin was Hipp11 (H11), which is located between *Eif4enif1* and *Drg1*. Expression of the *hPAX5* transgene is controlled by a third-generation tetracycline-response element. The H11*-*pTRE(3G)*-hPAX5* donor plasmid, in which the pTRE(3G)*-hPAX5* cassette was flanked by 2 H11 homologous arms, was used as a template for CRISPR/Cas9-mediated homologous recombination. sgRNA was designed to target the H11 locus with the sequence 5′-CTGAGCCAACAGTGGTAGTAAGG-3′. Cas9 mRNAs, sgRNA, and donor were co-microinjected into zygotes. After microinjection and embryo transfer, we obtained 6 pups. Primers for genotyping are listed in Supplemental Table 4.

### Immunohistochemical scoring of PAX5 expression.

PAX5 immunoreactivity was evaluated based primarily on nuclear staining. Staining intensity was graded as -, 0; +, 1; ++, 2; and +++, 3, while the staining extent was graded as <10%, 0; 10%–40%, 1; 40%–70%, 2; and >70%, 3. The final immunoreactivity score was calculated by multiplying the intensity score by the extent score. Based on this scoring system, PAX5 expression levels were categorized into 3 groups: negative (<0.1), low (0.1–3.9), and high (4.0–9.0).

### Orthotopic prostate tumor models.

A total of 5 × 10^5^ RM-1 cells expressing empty or PAX5 in 20 μL PBS containing 50% Matrigel were injected into the dorsal prostate lobe of 8-week-old male C57BL/6 mice (SPF Biotechnology Co., Ltd., Beijing, China). Two weeks after injection, the mice were sacrificed and lung metastases were examined.

### Cisplatin and etoposide administration.

Eight weeks after doxycycline administration, CC10-rtTA TetO-*hEGFR*^ex19del/T790M^ (EC) mice and CC10-rtTA TetO-*hEGFR*^ex19del/T790M^ TetO-*hPAX5* (EPC) mice, as well as RP and RPPa mice at 8 months postinfection with adenovirus-CMV-Cre, were used for treatment studies. CC10-rtTA TetO-*hEGFR*^ex19del/T790M^ transgenic mice were provided by Liang Chen (Jinan University, Guangzhou, China). Mice were treated with either a control (saline) or a combination of cisplatin and etoposide (E/P). Cisplatin (5 mg/kg, intraperitoneally) was administered on day 1, followed by etoposide (8 mg/kg, intraperitoneally) on days 1–3. One week was defined as 1 treatment cycle, and mice received 2 cycles of E/P therapy.

### Lectin perfusion assay.

A total of 10^6^ LLC cells expressing empty vector or *PAX5* in 100 μL PBS were injected into the flank of 8-week-old female C57BL/6 mice. After 2 weeks of injection, primary tumors were removed. Another 3 weeks later, mice were injected through the tail vein with rhodamine-conjugated dextran at 100 mg/kg. Three hours later, mice were injected through the tail vein with FITC-lectin at 10 mg/kg. Ten minutes later, each mouse was anesthetized and perfused with saline through the left ventricle. Lung tissues were cryosectioned into 5 μm and examined by fluorescence microscopy for vascular leakage.

### 3D fibrin gel bead sprouting assay in vitro.

The in vitro sprouting assay using 3D fibrin gel was performed as described previously (75) with minor modifications. In brief, HMVECs were suspended with Cytodex 3 microcarriers at a concentration of 400–450 cells per bead in 1 mL of DMEM growth medium, incubated for 4 hours at 37°C with gentle shaking every 20 min, transferred to a T25 tissue culture flask, and incubated overnight in 5 mL of DMEM at 37 °C and 5% CO_2_. The following day, the cell-coated beads were resuspended in 3 mg/mL of fibrinogen, received 0.625 U/mL of thrombin, and were placed in each well of 24-well plate. The fibrinogen/bead solution was allowed to clot for 5 min at room temperature before it was placed at 37°C and 5% CO_2_ for 15 min. We added 1 mL of CM to each well slowly (dropwise). Sprouting of HMVECs was monitored for 3 days. The number of primary sprouts and branches, and the total sprout length (cumulative length of primary sprouts and branches per spheroid), were quantitated using NIH Image.

### Bevacizumab and ZM323881 HCl treatment.

RP mice that received CMV-Cre viral infection were randomized into 2 groups. At 6 months after viral infection, vehicle (saline) or bevacizumab (10 mg/kg) was administered twice weekly via intraperitoneal injection for a duration of 1 month.

For mice bearing RM-1 tumors, bevacizumab (10 mg/kg) was administered 3 days after injection via intraperitoneal injection twice weekly. ZM323881 HCl (20 mg/kg) was administrated via intragastric administration daily. The treatment was continued until the end of the assay.

### CRISPR/Cas9-mediated PAX5 promoter deletion.

To establish a stable H1155 cell line with promoter deletion in the *PAX5* gene using CRISPR/Cas9 technique, we designed 2 sgRNAs specific for the *PAX5* promoter region from https://www.zlab.bio/resources (Supplemental Table 4). DNA oligonucleotides were synthesized and ligated into BbsI-digested pX458 plasmids expressing Cas9-sgRNA. The sg*PAX5* plasmids were transfected into H1155 cells. The cells expressing GFP were separated by flow cytometry after 24-hour transfection and cultured in a 96-well plate at 1 cell/well.

### ChIP-seq and analysis.

Cells were cross-linked with 1% formaldehyde for 10 min at room temperature. Cross-linking was terminated by 0.125 M glycine for 5 min. Cells were subsequently washed thrice with ice-cold PBS, collected in 15 mL tubes, and pelleted by centrifugation at 1,900 × *g* for 10 min at 4°C. Pellets were resuspended in 750 μL of sonication buffer (100 mM NaCl, 0.1% SDS, 5 mM EDTA pH 8.0, 100 mM Tris-HCl pH 8.0, 1% Triton X-100) and sonicated to obtain fragments (100–500 bp) with SONICS ultrasonic processor. Immunoprecipitation was performed with Anti-FLAG M2 Affinity Gel, overnight at 4°C with rotation. After elution and reversal cross-linking, DNA was purified and analyzed by next-generation sequencing.

Quality control was performed using FastQC v0.11.8. Clean reads were mapped to the reference genome GRCh38 using Bowtie2.3.5. For each sample, ChIP peaks were detected using MACS2. BigWig files were generated by deepTools. ChIP-seq tracks were visualized in IGV.

For ChIP-qPCR, the purified DNA was used as the template for SYBR Green–based qPCR analysis. ChIP signals were normalized to the input DNA, and the relative fold-enrichment was calculated against IgG controls. Primers used are listed in Supplemental Table 4.

### EMSA.

Nuclear proteins from A549 cells stably expressing PAX5-FLAG were extracted using a NucBuster protein extraction kit according to the manufacturer’s instructions. Double-stranded oligonucleotides corresponding to the potential PAX5 binding sites were end-labeled with biotin. EMSA was performed as described previously (76).

### Statistics.

Data are shown as mean ± SD unless otherwise indicated. All data points are derived from biological replicates, and *n* represents the number of independent biological samples. For histological analyses, 3–5 fields per mouse were averaged to yield a single value for that animal. Comparisons between 2 independent groups were performed using unpaired, 2-tailed Student’s *t* test. When the assumption of normality was not met, a 2-tailed Mann-Whitney *U* test was applied instead. For comparisons involving more than 2 groups, 1-way ANOVA was used, followed by Tukey’s or Dunnett’s multiple comparisons test as appropriate. In 2-factor experiments, 2-way ANOVA was conducted, and Šídák’s multiple comparisons test was used for pairwise comparisons of interest. A *P* < 0.05 was considered statistically significant. All statistical analyses were performed using GraphPad Prism (version 9.5.1).

### Study approval.

All animal procedures were approved by the Animal Care and Use Committee at Tianjin Medical University and conform to the legal mandates and national guidelines for the care and maintenance of laboratory animals. The use of all human lung cancer tissues was approved by the Institutional Review Board of Tianjin Medical University Cancer Hospital, and the use of all human prostate cancer tissues was approved by the Institutional Review Board of Tianjin Medical University Second Hospital. Written informed consent was obtained from all patients, and samples were deidentified prior to analysis.

### Data availability.

All data values are provided in the Supporting Data Values file. The raw sequencing data (RNA-seq, scRNA-seq, microarray, and ChIP-seq) have been deposited in the National Center for Biotechnology Information Gene Expression Omnibus under the accession numbers listed in Supplemental Table 5.

## Author contributions

AW, YH, XY, JL, LW, Y Jin, ZM, LST, and ZL conceived the project, analyzed and interpreted the data, and wrote the manuscript. WL performed the bioinformatics. YH, Y Jiang, and XC contributed to the data collection. Jun Yan and WG performed pathology review and analysis. ZS, ZZ, and YN contributed to data analysis and material support. LG, BD, and JDM contributed to the acquisition of data and reviewed/edited the manuscript.

## Conflict of interest

The authors have declared that no conflict of interest exists.

## Funding support

This work is the result of NIH funding, in whole or in part, and is subject to the NIH Public Access Policy. Through acceptance of this federal funding, the NIH has been given a right to make the work publicly available in PubMed Central.

- The National Natural Science Foundation of China (grants U25A20109 and 82573173 to ZL, 82573216 to ZM, 82273119 to ZZ).
- The Interdisciplinary Research Project of Hangzhou Normal University (2024JCXK03 to ZL).
- The NIH (grants R01CA208620 to LST, U24CA213274, P50CA070907, and U01CA213338 to JDM).
- The Cancer Prevention and Research Institute of Texas (grant RP160307 to LST).

## Supplementary Material

Supplemental data

Unedited blot and gel images

Supporting data values

## Version 1 07/23/2026

In-Press Preview

## Version 2 09/15/2026

Electronic publication
