# Supplementary material for: PAX5 modulates vascularization and contributes to neuroendocrine lineage transition during carcinoma progression
Source: J Clin Invest. 2026 Jul 23;136(18):e190596. doi: 10.1172/JCI190596 (PMC13574158; doi:10.1172/JCI190596)
Supplement: Supplementary file 1 [file jci-136-190596-s268.pdf]

Supplemental Materials for

PAX5 modulates vascularization and contributes to neuroendocrine lineage transition during carcinoma progression

Ailing Wu, Yujie Hao, Xuemiao Yan, Junrong Liu, Lin Wang, Yan Jin, Wenxu Liu, Xiyue Chen, Yuan Jiang, Luc Girard, Zhiqun Shang, Jun Yan, Zhenfa Zhang, Wenchen Gong, Yuanjie Niu, Benjamin J. Drapkin, John D. Minna, Lance S. Terada, Zhenyi Ma, Zhe Liu

Supplemental Methods

Xenograft and Syngeneic tumor models

10^6 A549 cells expressing empty vector or *PAX5* were mixed with 5×10^5 human lung CAFs in 100 µL PBS containing Matrigel (1:1 vol/vol) and were injected into the flank of 8-week-old female BALB/c Nude mice. Eight weeks after injection, the mice were sacrificed and analyzed.

10^6 H1155 cells or *PAX5*-KO-H1155 cells were mixed with 5×10^5 human lung CAFs in 100 µL PBS containing 50% Matrigel and were injected into the flank of 8-week-old female NOD-SCID mice. All mice were sacrificed when the largest tumor reached 500 mm^3 and tumors were fixed and embedded in paraffin for further analyses.

10^6 Lewis lung carcinoma (LLC) cells expressing empty vector or *PAX5* in 100 µL saline was injected into the flank of 8-week-old female C57BL/6 mice. All mice were sacrificed when the largest tumor reached 1000 mm^3 and tumors were fixed and embedded in paraffin for further analyses.

Soft agar colony formation assay

BEAS-2B, 16HBE, and PC-9 cells were embedded in 0.35% soft agar layered over a 0.6% agar base in 6-well plates. Following a 2-3 weeks culture period, colonies were stained with crystal violet and imaged.

Supplemental Figures

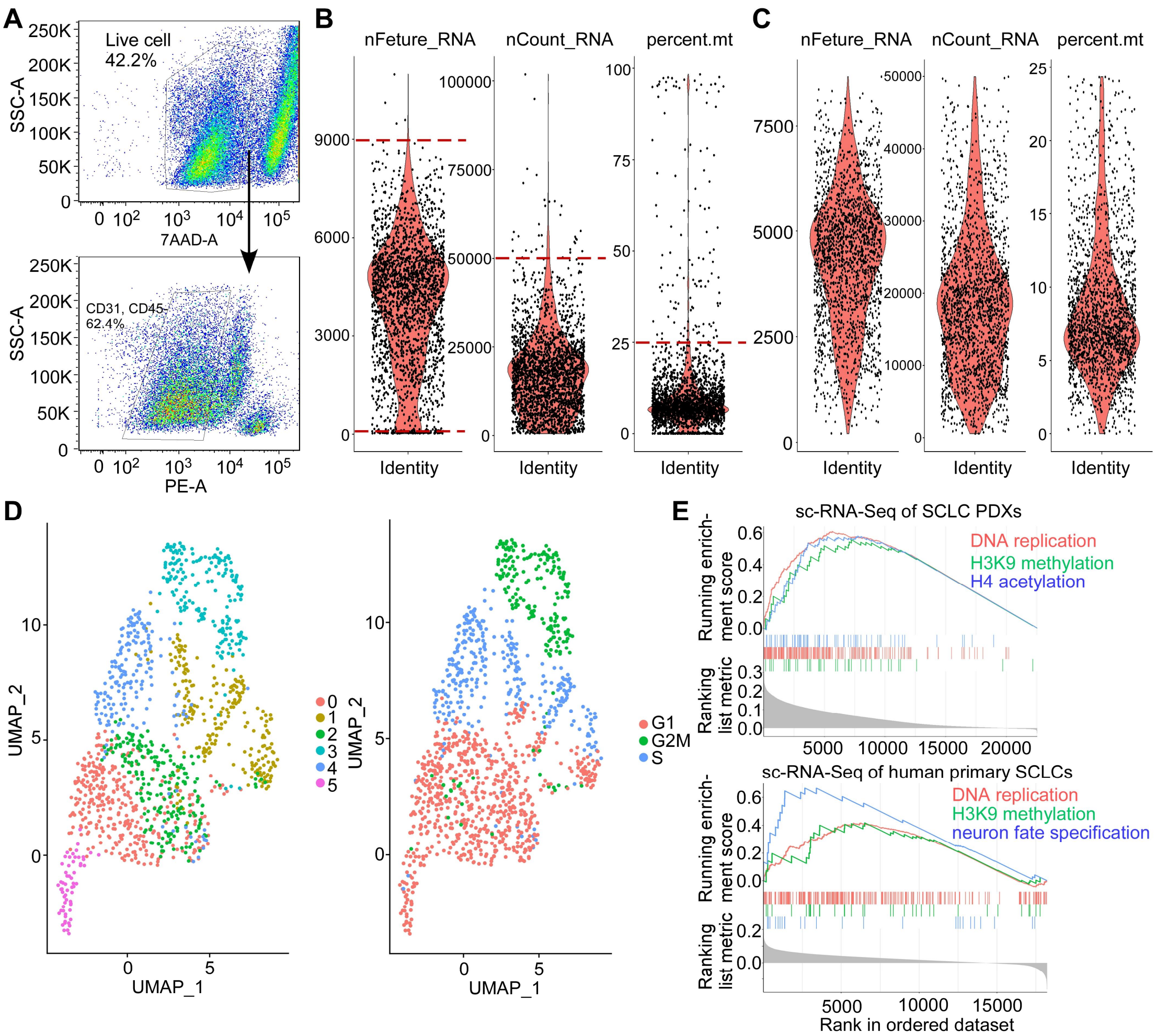

Supplemental Figure 1. scRNA-seq confirms *PAX5* signaling enrichment. (A-C) Flow sorting and quality control of single cell RNA sequencing (scRNA-seq) data of tumor cells. (**A**) DAPI, CD45 and CD31 triple negative cells were sorted for scRNA-seq. Distribution of the number of total genes (nFeature_RNA) and total molecules (nCount_RNA) detected per cell before (**B**) and after (**C**) quality control. Cells with gene numbers at between 200 and 9000 genes were retained for further analysis (red dashed lines). Distribution of percentage of mitochondria genes (percent.mt) per cell before (**B**) and after (**C**) quality control. Cells with less than 25% mitochondrial genes were selected for further analysis (red dashed lines). **(D)** NE-SCLC clusters 0, 1, 2, 3 and 4 were classified by expression of cell cycle-related genes. **(E)** Gene set enrichment

analysis (GSEA) shows the enriched pathways positively correlated with *PAX5* expression in published scRNA-seq of SCLC circulating tumor cell-derived PDXs (top panel) and published scRNA-seq of human primary SCLCs (bottom panel).

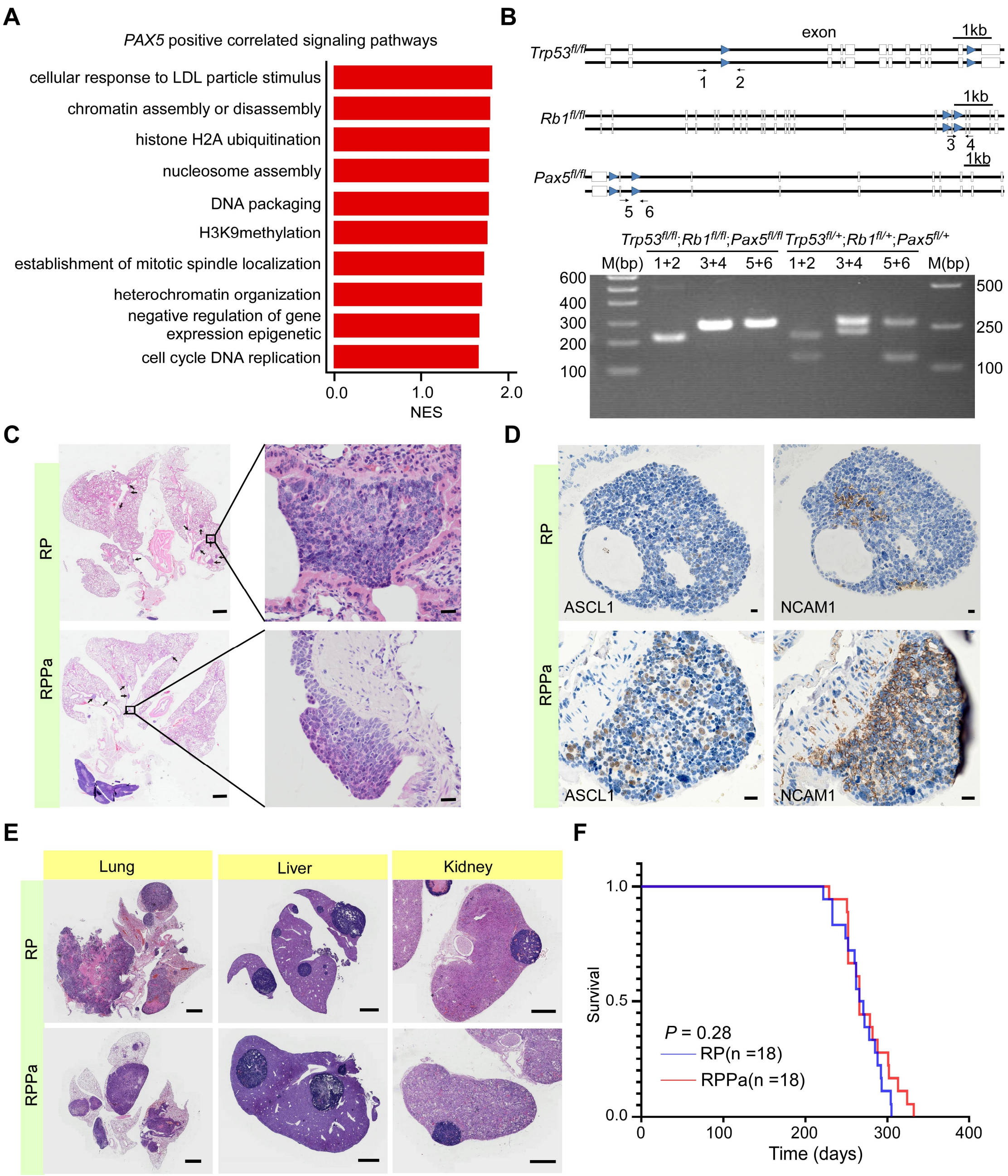

Supplemental Figure 2. Generation of *PAX5*-driven SCLC models. (A) GSEA using RNA-seq data of 79 SCLC cell lines (generated by Minna's group) and 51 PDXs (generated by Drapkin's group) showing the signaling pathways positively correlated with *PAX5*, with normalized enrichment scores (NES) indicating the degree of enrichment. **(B)** Schematic diagrams of *Trp53*, *Rb1*, and *Pax5* conditional alleles are shown in the top panels. Triangles represent LoxP sites. Arrows indicate primers used

in genotyping. Bottom panel shows homozygous and heterozygous genotyping. **(C** and **D)** Representative H&E staining **(C)** and immunostaining **(D)** images of tumors developed in RP and RPPa mice at 5 months post infection with adenovirus carrying CMV-Cre (*n* = 3 independent mice per group). Scale bars, 2 mm (left panel of C) and 20 μm (right panel of C and D). **(E)** Representative images of H&E staining of primary lung tumor nodules and metastatic nodules in the livers and kidneys, developed in RP and RPPa mice at 8 months post infection of adenovirus carrying CMV-Cre (*n* = 5 independent mice per group). Scale bar, 2 mm. **(F)** Survival rates of RP and RPPa mice.

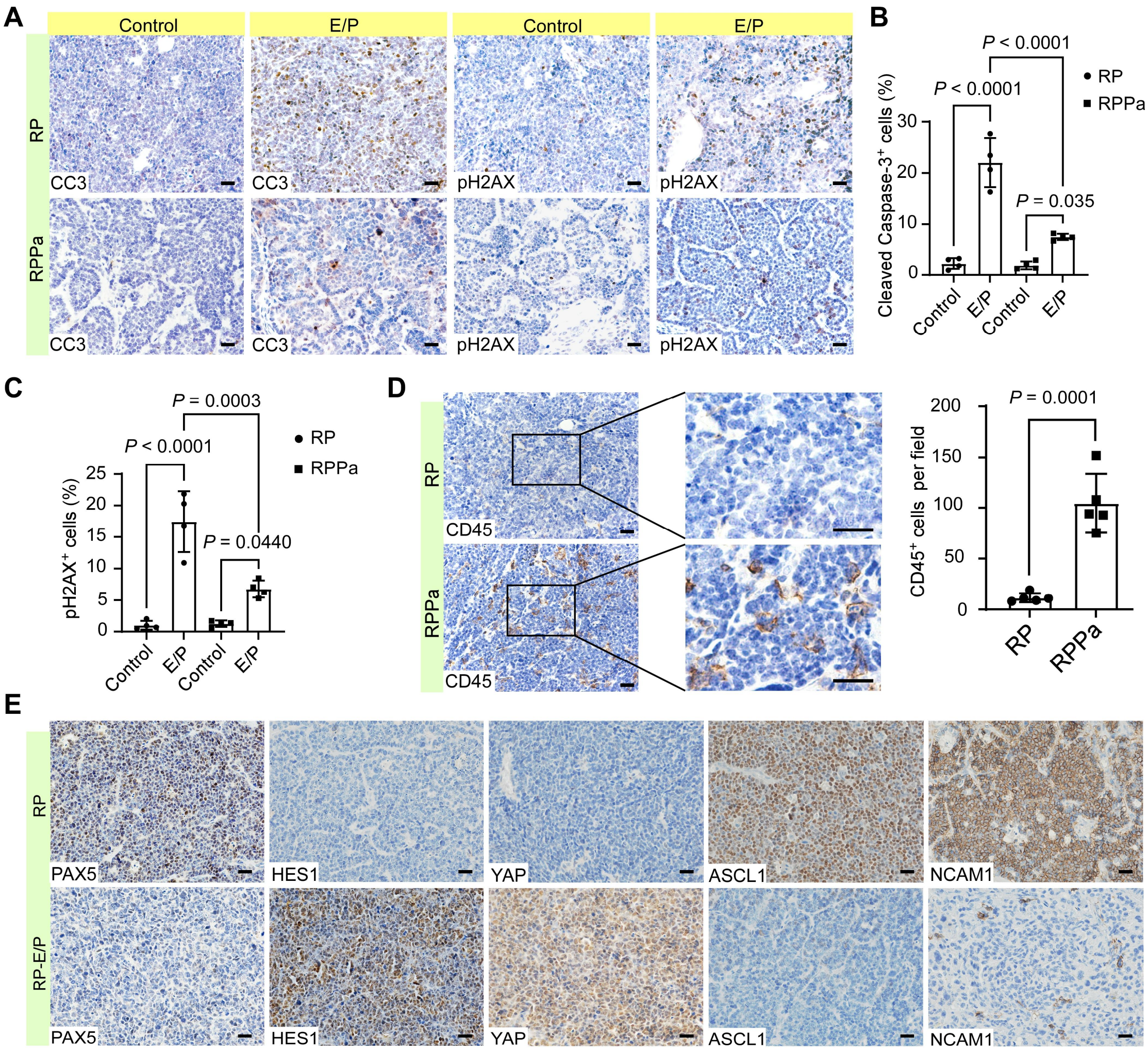

Supplemental Figure 3. PAX5 deficiency attenuates chemotherapy-induced apoptosis and DNA damage in SCLC. (A-C) Representative images of cleaved caspase-3 (CC3) and pH2AX immunostaining in tumors from RP and RPPa mice treated with control (saline) or a combination of etoposide and cisplatin (E/P) (n = 4 independent mice per group), with the corresponding quantification of CC3 (**B**) and pH2AX (**C**). Scale bar, 20 µm. Data are presented as mean ± SD (n = 4 mice per group); two-way ANOVA followed by Šídák's multiple comparisons test for the indicated pairwise comparisons. **(D)** Representative immunostaining of tumors developed in RP mice and RPPa mice (n = 5 independent mice per group). Scale bar, 20 µm. Data are

shown as mean ± SD (n = 5 mice per group); unpaired, two-tailed Student's t-test. **(E)** Immunostaining in tumors treated with either saline or E/P in RP and RPPa mice. Scale bar, 20 μm.

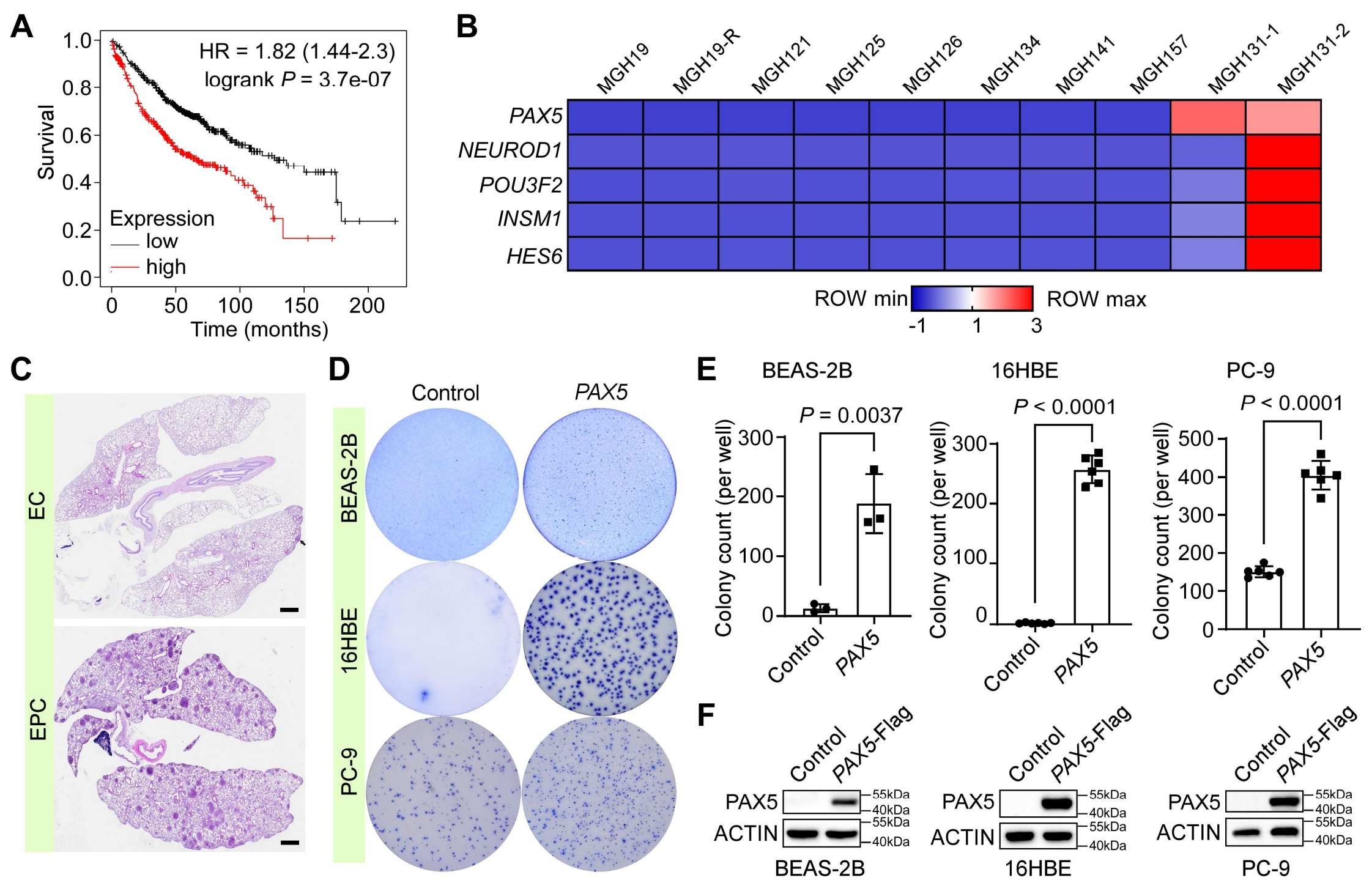

Supplemental Figure 4. PAX5 promotes malignant transformation of lung adenocarcinoma. (A) Kaplan-Meier survival rates for 1155 subjects with lung adenocarcinoma with low (0-4.0 staining scores, black lines, *n* = 753), versus high (4.1-8.0 staining scores, red lines; *n* = 402) *PAX5* expression were compared. **(B)** Expression of *PAX5, NEUROD1, POU3F2, INSM1, HES6* in cell lines derived from biopsies of resistant lung adenocarcinomas that maintained adenocarcinoma histology (MGH119, MGH119-R, MGH121, MGH125, MGH126, MGH134, MGH141, MGH157) and that transformed to SCLC (MGH131-1 and MGH131-2, two biopsies derived from the same patient but taken several months apart). **(C)** Representative H&E staining and scanning images of lung tumor nodules from CC10-rtTA;TetO-*hEGFR*$^{ex19del/T790M}$ (EC) and CC10-rtTA;TetO-*hEGFR*$^{ex19del/T790M}$;TetO-*hPAX5* (EPC) mice at 1 month post doxycycline treatment (*n* = 4 independent mice per group). Scale bar, 2mm. **(D-F)** Soft agar colony formation assay in BEAS-2B, 16HBE, and PC-9 cells. Representative

crystal violet staining (**D**) and quantification of colony numbers (**E**) are shown. Data are mean ± SD (BEAS-2B: *n* = 3 technical replicates per group; 16HBE and PC-9: *n* = 6 technical replicates per group), representative of three independent experiments; unpaired, two-tailed Student's *t*-test. **(F)** Western blot analysis confirming PAX5 overexpression in BEAS-2B, 16HBE, and PC-9 cells.

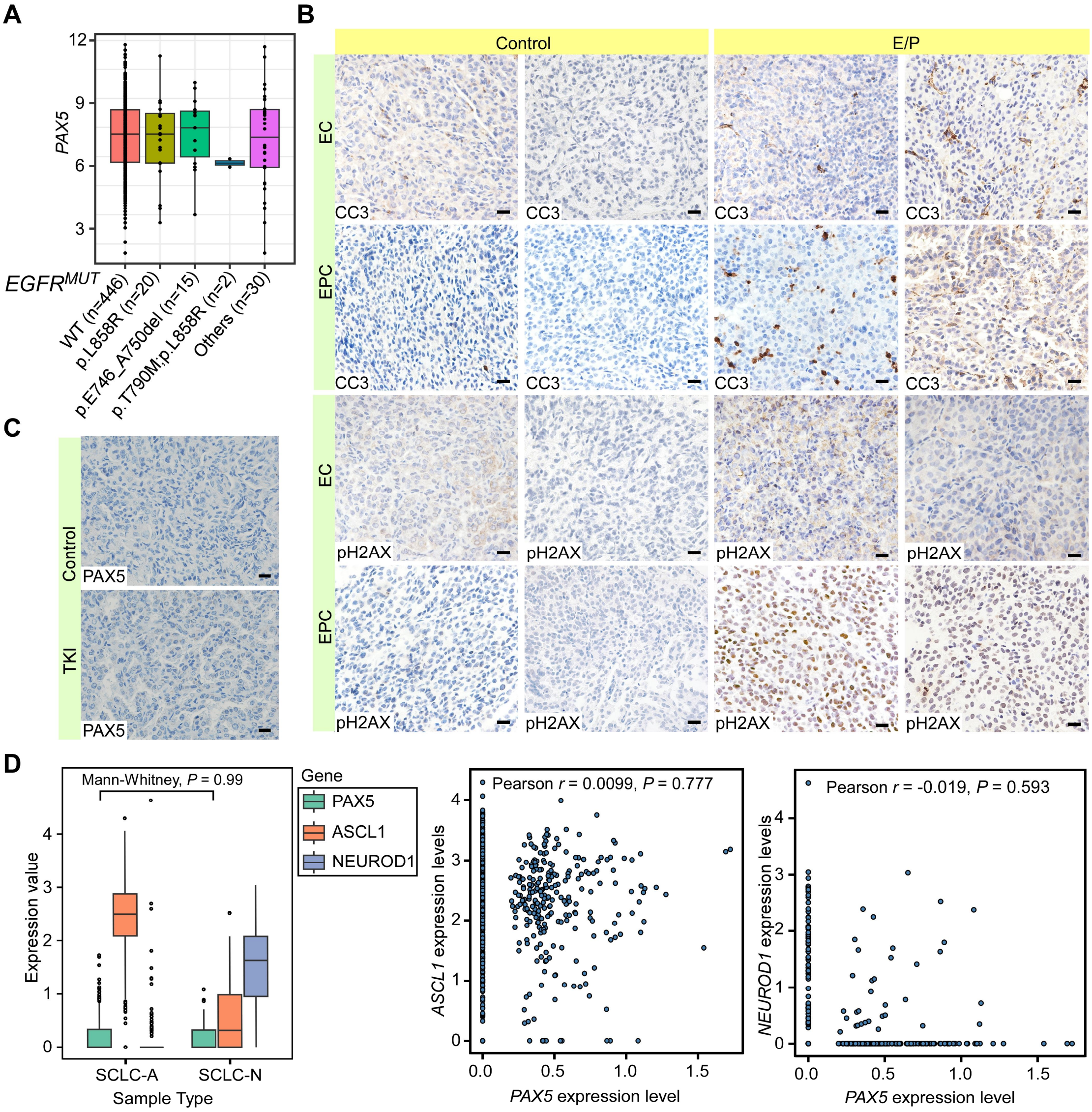

Supplemental Figure 5. PAX5 overexpression enhances chemosensitivity in lung adenocarcinoma. **(A)** Analysis of TCGA data sets for the relationship between *PAX5* expression and *EGFR* mutation. Data are presented as boxplots. **(B)** CC3 and pH2AX immunostaining in tumors from EC and EPC mice treated with either saline or E/P. Images from two mice per group are shown. Scale bar, 20 µm. **(C)** Immunostaining in EC tumors treated with erlotinib for 2 weeks. Scale bar, 20 µm. **(D)** Analysis of scRNA-seq data (left panel) and established different subtype of SCLC lines (middle and right panel) for the correlation between *PAX5* and *ASCL1* or *NEUROD1*.

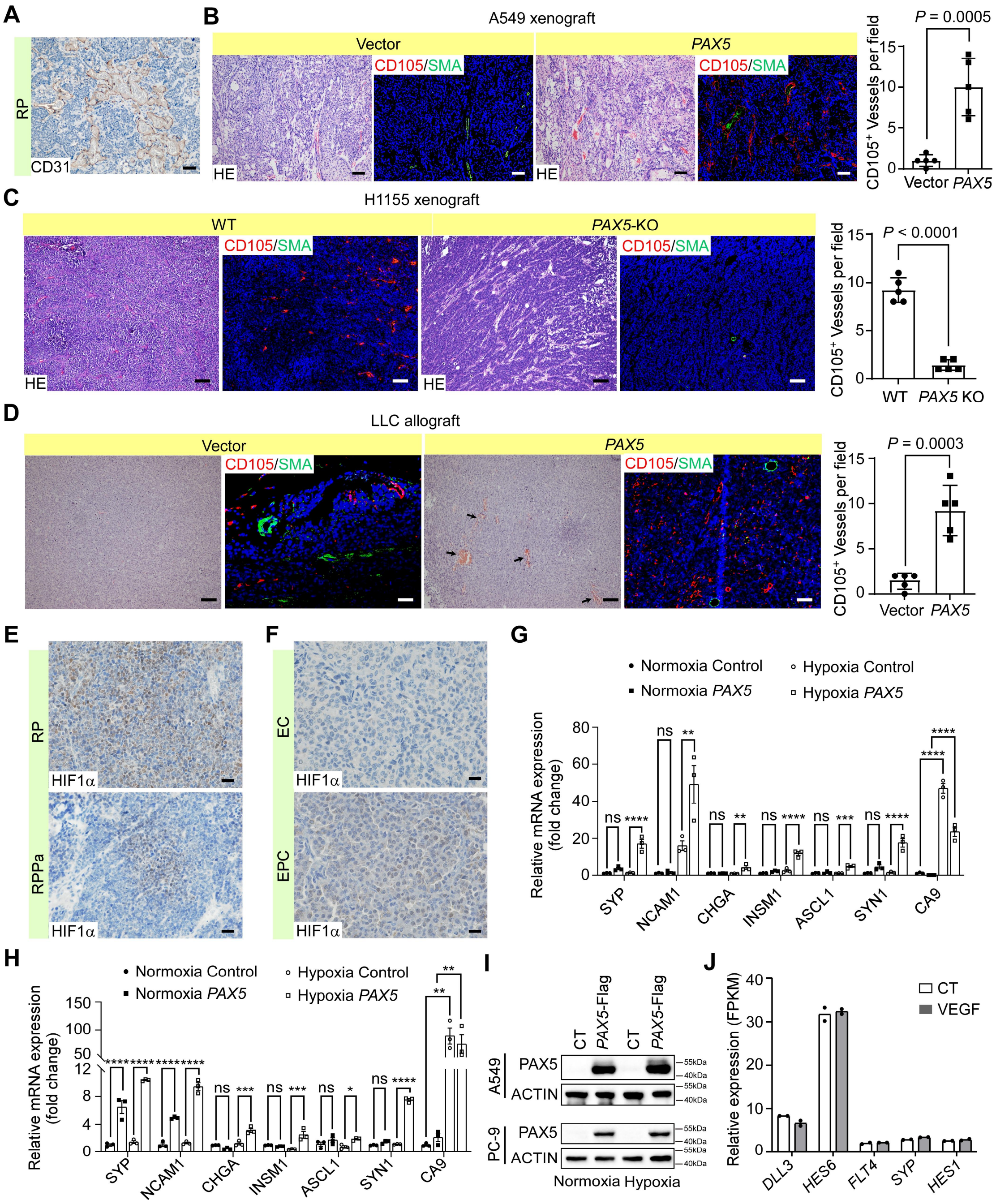

Supplemental Figure 6. PAX5 promotes angiogenesis and hypoxia-regulated NE gene expression. (**A**) Immunostaining in RP tumors**.** Scale bar, 50 μm. **(B** and **C)** Representative H&E staining and immunofluorescence images for CD105 and α-SMA in subcutaneous A549 (**B**) and H1155 (**C**) tumors (left panels, n = 5 independent mice per group), with the corresponding quantification of vessels (right panel). Scale bar, 100 μm. Data are mean ± SD (*n* = 5 mice per group); unpaired, two-tailed Student's *t*-

test. (**D**) Representative H&E staining and immunofluorescence images for CD105 and α-SMA in subcutaneous tumors derived from vector or *PAX5*-expressing LLC cells in C57BL/6 mice (left panels, $n = 5$ independent mice per group), with the corresponding quantification of vessels (right panel). Arrows point to large irregular vessels. Scale bars are 100 μm. Data are mean ± SD ($n = 5$ independent mice per group); unpaired, two-tailed Student's *t*-test. **(E** and **F)** Immunostaining in RP and RPPa tumors (**E**) and EC and EPC tumors (**F**)**.** Scale bar, 20 μm. **(G** and **H)** qPCR analysis of NE gene expression in A549 (**G**) and PC-9 (**H**) cells expressing Control or *PAX5* under normoxia and hypoxia (1% O_2; 6 days for A549 and 5 days for PC-9). Total RNA was then extracted for analysis. Data are mean ± SD of technical triplicates from one representative of three independent experiments; two-way ANOVA followed by Šídák's multiple comparisons test for the indicated pairwise comparisons. ns, no significant, * $p < 0.05$, ** $p < 0.01$, *** $p < 0.001$, **** $p < 0.0001$. **(I)** Western blot analysis of PAX5 expression in the indicated cells. **(J)** RNA-seq analysis of A549 cells treated with VEGF. Gene expression values are normalized and presented in FPKM. Data are from two independent biological replicates.

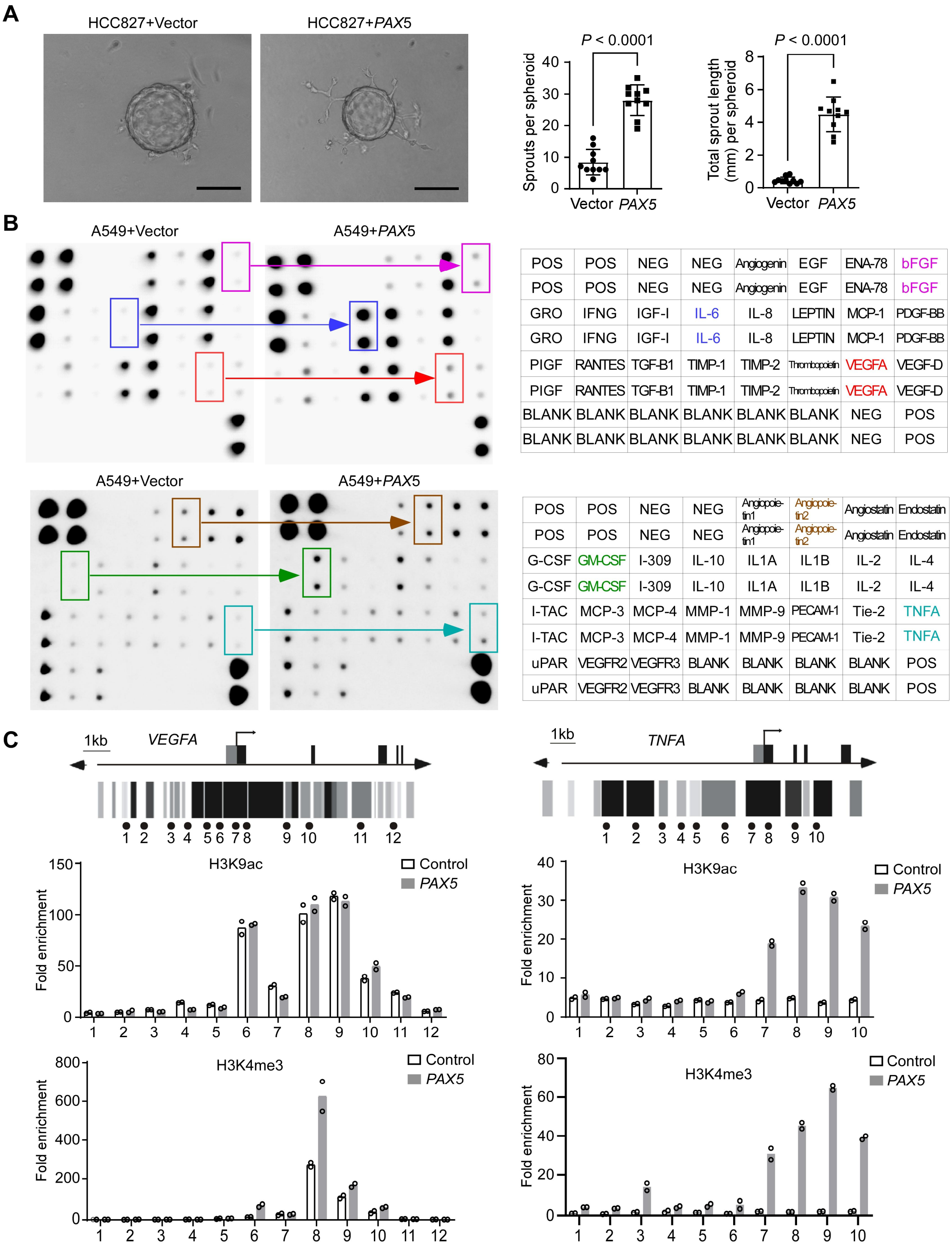

Supplemental Figure 7. PAX5 induces an angiogenic secretome and epigenetic activation of angiogenic genes. (A) CM collected from HCC827 human lung adenocarcinoma cells transduced with *PAX5* were added into HMVECs spheroid culture. Representative micrographs showing HMVECs spheroid sprouting (left panels,

n = 10 independent spheroids per group). Scale bar, 200 μm. Sprout number and length were quantified (right panels). Data are shown as mean ± SD (*n* = 10 spheroids); unpaired, two-tailed Student's *t*-test. **(B)** Images of protein array of angiogenesis related factors in CM. CM from A549 cells transduced with empty vector or *PAX5* were used for this assay. Each factor was analyzed in duplicate, representative of two independent experiments. **(C)** The distribution of H3K9ac and H3K4me3 within genomic loci of *VEGFA* and *TNFA* were examined using chromatin immunoprecipitation (ChIP). For each gene, DNase I hypersensitive sites (ENCODE, vertical lines), in the ± 5-kb region of the transcriptional starting site were analysed. ChIP was performed in control and *PAX5*-expressing A549 cells. Two technical replicates from one representative experiment of three independent experiments are shown.

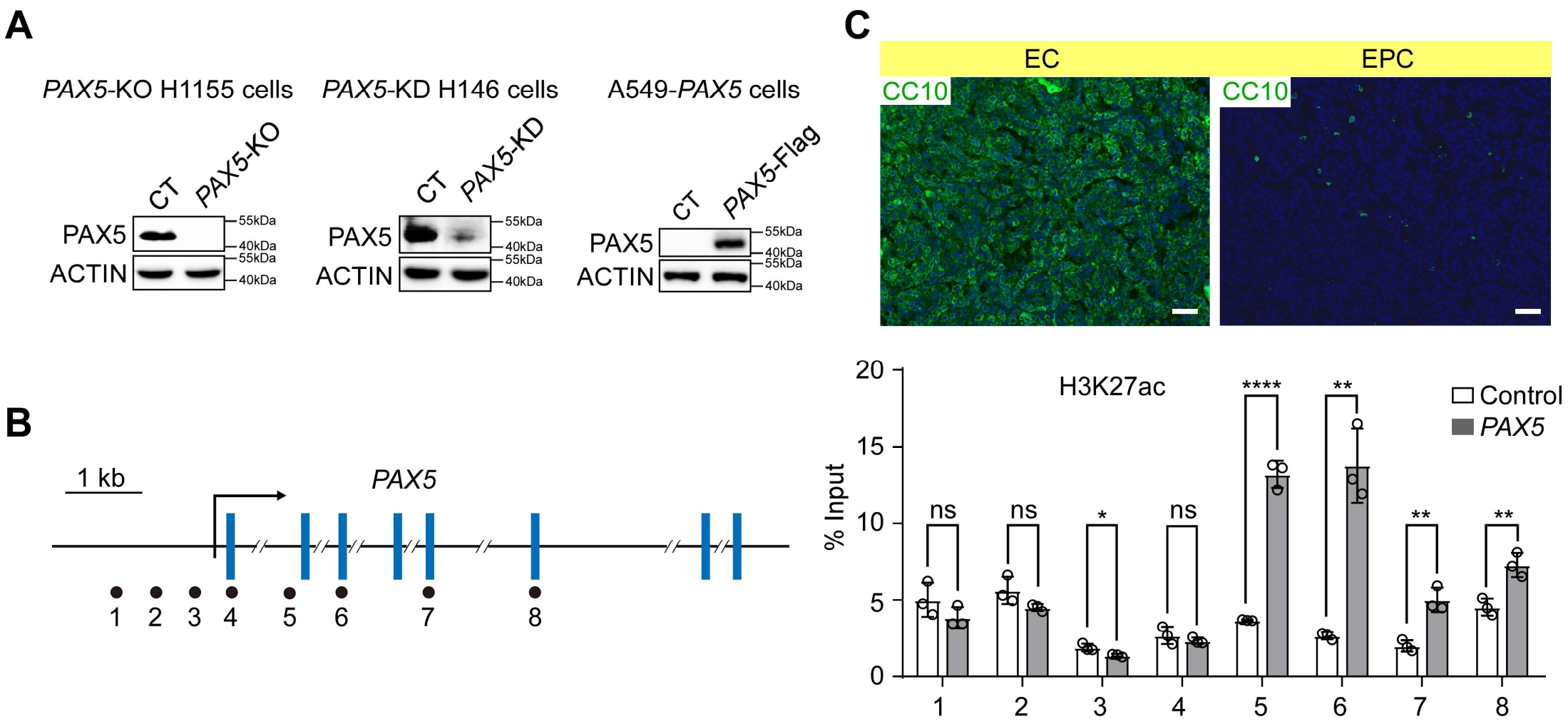

Supplemental Figure 8. *PAX5* drives its own expression through epigenetic activation. (A) Western blot analysis confirming the knockout, knockdown, and overexpression of PAX5 in H1155, H146, and A549 cell lines. (**B**) ChIP-qPCR showing H3K27ac occupancy at the endogenous *PAX5* locus, presented as % input. A representative from three independent experiments is shown. Data are mean ± SD of three technical replicates; unpaired, two-tailed Student's *t*-test. ns, no significant, * $p < 0.05$, ** $p < 0.01$, **** $p < 0.0001$. **(C)** Immunostaning of PAX5 in EC and EPC tumors. Scale bar, 50μm.

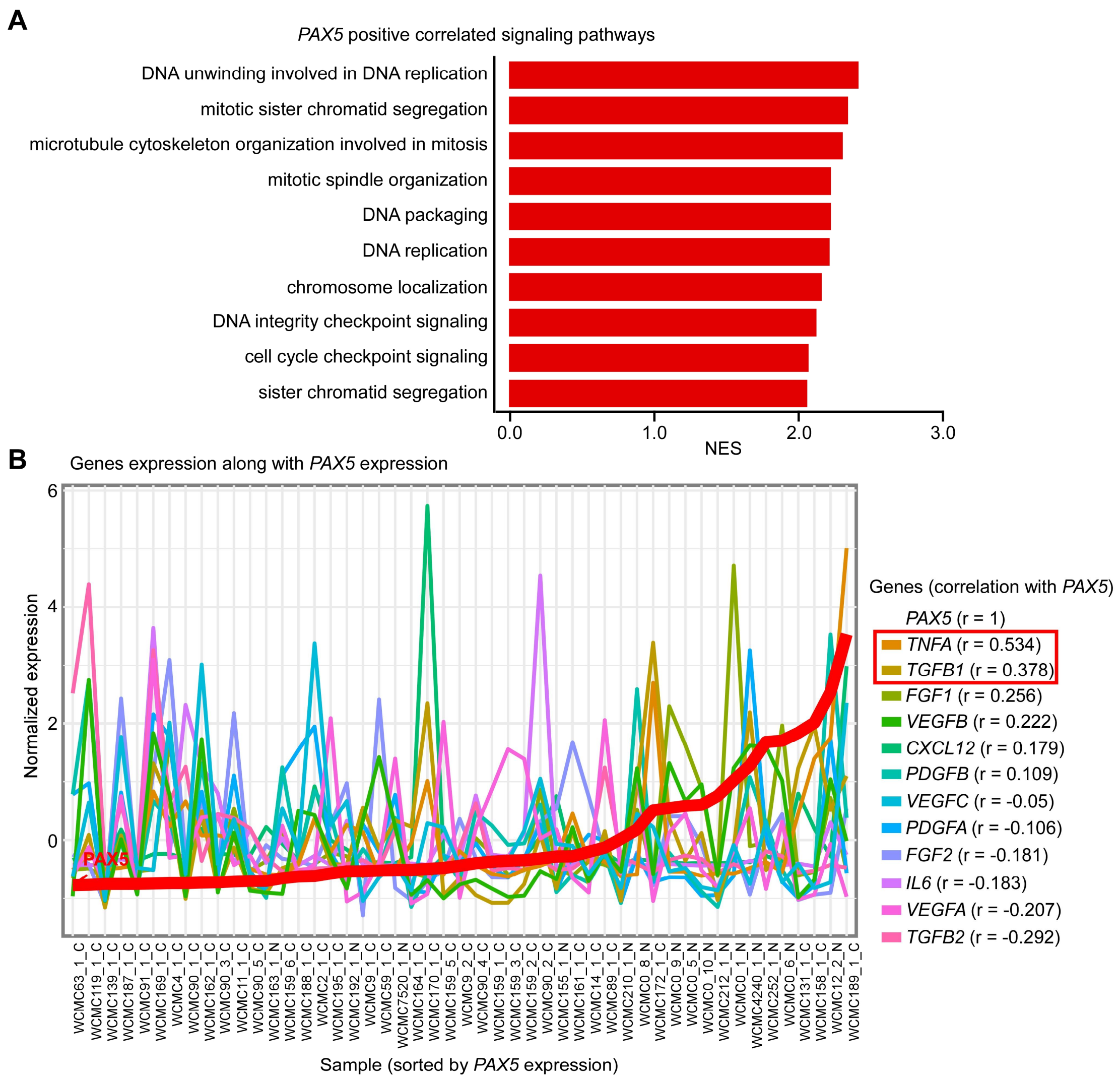

Supplemental Figure 9. *PAX5*-correlated signaling and GO enrichment in NEPC. **(A)** GSEA showing the signaling pathways positively correlated with *PAX5*, with normalized enrichment scores (NES) indicating the degree of enrichment. **(B)** GSEA-GO analysis on the RNA-seq dataset of 15 NEPCs.

Supplemental Table 1. Clinical characteristics of human primary SCLC analyzed by scRNA-seq.

Patient number	Gender	Age	Procedure type	Tissue type	Site	Smoking status	Histology	Treatment status	EG FR	K-RAS	Vital Status
SCLC	Male	72	Resection	Primary	Lung	Current	Small cell lung cancer	Naïve	WT	WT	Alive

Supplemental Table 2. Feature summary of serum for sprouting assay from patients.

NO	Sex	Age	Somking	Histology	Pathology	TNM	Stages
1	Female	59	NO	LAD	PD	T1N0M0	I
2	Female	54	YES	LAD	PD	T2N2M1	IV
3	Male	56	YES	LAD	na	T1N2M1	IV
4	Female	49	YES	LAD	PD	T1N3M1	IV
5	Male	55	YES	LAD	na	T3N0M1	IV
6	Male	60	YES	LAD	na	T2N2M0	III
7	Female	60	NO	LAD	PD	T1N2M0	III
8	Female	52	NO	LAD	WD	T1N1M0	II
9	Male	61	YES	SCLC	PD	T1N3M1	IV
10	Male	60	YES	SCLC	PD	T1N2M1	IV
11	Male	69	YES	SCLC	na	T1N2M1	IV
12	Male	49	YES	SCLC	PD	T1N2M1	IV
13	Male	57	YES	SCLC	na	T1N0M0	I
14	Male	55	YES	SCLC	PD	T2N3M1	IV
15	Male	50	YES	SCLC	na	T1N0M0	I
16	Female	68	NO	SCLC	PD	T4N2M1	IV

LAD, lung adenocarcinoma; SCLC, small cell lung cancer; PD, poorly differentiated; WD, well differentiated.

Supplemental Table 3. Reagent or Resource.

REAGENT or RESOURCE	SOURCE	IDENTIFIER
Rabbit anti-PAX5	Abcam	Cat# 109443; RRID: AB_10862070
Rabbit anti-SYP	Sigma	Cat# SAB4502906; RRID: AB_10746692
Rabbit anti-CHGA	Abcam	Cat# 15160; RRID: AB_301704
Mouse anti-NeuroD1	Abcam	Cat# 60704; RRID: AB_943491
Rabbit-anti-KRT7	Abcam	Cat# 181598; RRID: AB_2783822
Rabbit anti-ASCL1	Abcam	Cat# 211327; RRID: AB_2924270
Rabbit anti-NCAM1	Abcam	Cat# 220360; RRID: AB_2927664
Rabbit anti-pH2AX	Abcam	Cat# 81299; RRID: AB_1640564
Rabbit anti-YAP	AB clonal	Cat# A1002; RRID: AB_2757539
Rabbit anti-Cleaved Caspase-3	Cell Signaling Technology	Cat# 9664; RRID: AB_2070042
Rabbit anti-Ki-67	Cell Signaling Technology	Cat# 12202; RRID: AB_2620142
Rabbit anti-Vimentin	Cell Signaling Technology	Cat# 5741; RRID: AB_10695459
Rabbit anti-CD74	Cell Signaling Technology	Cat# 77274; RRID: AB_2799893
Mouse anti-SYP	Cell Signaling Technology	Cat# 9020; RRID: AB_2631095
Rabbit anti-α-SMA	Abcam	Cat# 15734; RRID: AB_443242
Mouse anti-CD105	Abcam	Cat# 11414; RRID: AB_298019
Rabbit anti-PAX5	Abcam	Cat# 15164; RRID: AB_2159682
PE anti-Human CD45 Monoclonal Antibodies	Sungenebiotech	Cat# H20451-09H
PE anti-Human CD31 Monoclonal Antibodies	Biolegend	Cat# 303106; RRID: AB_314332
DAPI Solution	BD Biosciences	Cat# 564907; RRID: AB_2869624
Mouse anti-Actin	Millipore	Cat# MAB1501; RRID: AB_2223041
Anti-FLAG M2 affinity gels	Sigma-Aldrich	Cat# A2220; RRID: AB_10063035
Goat anti-Rabbit IgG (H+L)-HRP Secondary Antibody	Bioworld Technology	Cat# BS13278; RRID: AB_2773728
Goat anti-Mouse IgG (H+L)-HRP Secondary Antibody	Thermo Fisher Scientific	Cat# 31430; RRID: AB_228307
DMEM medium	Gibco	Cat# C11995500BT

RPMI 1640	Gibco	Cat# C11875500BT
DMEM/F-12	Gibco	Cat# C11330500BT
Fetal Bovine Serum	Biological Industries	Cat# 04-001-1ACS
MEM NEAA (100×)	Gibco	Cat# 11140-050
Penicillin-Streptomycin-Glutamine (100X) Gibco™	Gibco	Cat# 15140-122
MACS Tissue Storage Solution	Miltenyi Biotec	Cat# 130-100-008
DNase I	Sigma-Aldrich	Cat# D4527
Red Blood Cell Lysis Buffer	Solarbio	Cat# R1010
Cell Strainer (70 µm)	Falcon	Cat# 352350
Dextran, Rhodamine B, 10,000MV, Neutral	Thermo Fisher Scientific	Cat# D1824
FITC-Lectin	Sigma-Aldrich	Cat# L9006
2,2,2-Tribromoethanol	Sigma-Aldrich	Cat# T48402
Cytodex® 3 Microcarrier Beads	Sigma-Aldrich	Cat# C3275
Fibrinogen from Human plasma	Sigma-Aldrich	Cat# F3879
Thrombin from Human plasma	Sigma-Aldrich	Cat# T6884
Bevacizumab Injection Avastin®	Roche	S20170035
ZM 323881 HCl	Selleck.cn	Cat# S2896
Phosphate Buffered Saline	Biological Industries	Cat# 02-024-1ACS
BbsI-HF	New England Biolabs	Cat# R3539
Quick CIP	New England Biolabs	Cat# M0525
T4 Polynucleotide Kinase	New England Biolabs	Cat# M0201
T4 DNA Ligase	New England Biolabs	Cat# M0202
Cisplatin	Selleck.cn	Cat# S1166
Etoposide	Selleck.cn	Cat# S1225
Protein A Sepharose CL-48	GE Healthcare	Cat# 17-0780-01
Ad-CMV-Cre	Vector Biolabs	Cat# 1045
Protease Inhibitor Cocktail Tables	Roche	Cat# 04693132001
4% paraformaldehyde	Solarbio	Cat# No. P1110
Tumor Cell Isolation Kit, human	Miltenyi Biotec	Cat# 130-108-339

NucBuster protein extraction kit	Novagen	Cat# 71183-3
Chromium Single Cell 3' Library & Gel Bead Kit v2	10x Genomics	PN-120267
QIAquick PCR Purification Kit	QIAGEN	Cat# No.28104
LightShift™ Chemiluminescent EMSA Kit	Thermo Fisher Scientific	Cat# 20148
Chemiluminescent Nucleic Acid Detection Module Kit	Thermo Fisher Scientific	Cat# 89880
Mouse Direct PCR Kit	bimake.cn	Cat# B40013
Lipofectamine® 3000 Transfection Kit	Thermo Fisher Scientific	Cat# L3000-015
HMVEC	Lonza	CC2505
H526	ATCC	CRL-5811TM
H146	ATCC	HTB-173
H69	ATCC	HTB-119
H82	ATCC	HTB-175
H460	ATCC	HTB-177
HCC827	ATCC	CRL-2868
H1155	ATCC	CRL-5818
A549	ATCC	CCL-185
LNCAP	ATCC	CRL-1740
LLC	ATCC	CRL-1642
RM-1	ATCC	CRL-3310TM
NIH3T3	ATCC	CRL-1658
CC10-rtTA;TetO-*hEGFR*$^{ex19del/T790M}$ mice	Gifts from Dr. Liang Chen	N/A
TetO-hPAX5 mice	This paper	N/A
Rb1$^{fl/fl}$*;Trp53*$^{fl/fl}$	Gifts from Dr. Hongbin Ji	N/A
Pax5$^{fl/fl}$	The Jackson Laboratory	28100
pSpCas9(BB)-2A-GFP (PX458)	Addgene	Cat# 48138
GraphPad Prism 9.5.1	GraphPad Software Inc.	http://www.graphpad.com
FlowJo	FlowJo Software	https://www.flowjo.com/
Fiji NIH	ImageJ	https://imagej.nih.gov/ij/docs/guide/146-2.html

Gene Set Enrichment Analysis (GSEA)	Broad Institute	http://software.broadinstitute.org/gsea/index.jsp
Kaplan-Meier Plotter	KMplot.com	http://kmplot.com
Cell Ranger (v3.1)	Cell Ranger Single Cell Software Suite	https://support.10xgenomics.com/single-cellgene-expression/software/pipelines/latest/what-is-cell-ranger
Seurat	Seurat R package	https://satijalab.org/seurat/
HOMER	Heinz et al., 2010	http://homer.ucsd.edu/homer/
Bowtie 2	Langmead and Salzberg, 2012	http://bowtie-bio.sourceforge.net/bowtie2/
MACS2	Zhang et al., 2008	https://github.com/taoliu/MACS
IGV (v2.9.2)	Integrative Genomics Viewer	https://software.broadinstitute.org/
Human genome GRCh38	N/A	ftp://ftp.ensembl.org/pub/release-94/
R v3.6.3	The R Project for Statistical Computing	http://www.r-project.org

Supplemental Table 4. Primers used in the study.

sgRNA sequences	
PAX5-sgRNA-1	ACTCCTGGCCCGCCGGACAATGG
PAX5-sgRNA-2	GACAGGTAGGACCGCGATACGGG
Primers for construction of luciferase reporters	
luci-*VEGFA*-F	GGGGTACCGTTCTCAGCTCCACAAACTTG
luci-*VEGFA*-R	CCGCTCGAGAGGGATAAAACCCGGATCAATGAATAT
luci-*TNFA*-F	GGGGTACCACAGCAATGGGTAGGAGAATG
luci-*TNFA*-R	CCGCTCGAGGTGCCAACAACTGCCTTTATATG
Primers for ChIP-qPCR	
VEGFA-1F	GTGGCTCAGAGAGGTTGAATAA
VEGFA-1R	GTAGGGCAGTGGAAAGTACAG
VEGFA-2F	GGATGGAGTGAAGGACAAGAC
VEGFA-2R	TGGAAAGAAGGTGAGGAGAGA
VEGFA-3F	AAGAAGGCCGAGTTGAGAAG
VEGFA-3R	GGAGCACAAGCTGTAGACAT
VEGFA-4F	AGATGTGGAGAGTTGGAGGA
VEGFA-4R	AGGATCCCTTGGCTACTTCT
VEGFA-5F	TGTTTGGGAGGTCAGAAATAGG
VEGFA-5R	TAAAGAGGGAATGGGCTTTGG
VEGFA-6F	CAATAGATCTGTGTGTCCCTCTC
VEGFA-6R	AGCCGTTCCCTCTTTGCTA
VEGFA-7F	CAAAGTGAGTGACCTGCTT
VEGFA-7R	TGTCTGTCTGTCTGTCCGT
VEGFA-8F	CCATGAACTTTCTGCTGTCTTG
VEGFA-8R	GCACGACCGCTTACCTT
VEGFA-9F	CTGTGACCTTGGCTGTCTC
VEGFA-9R	GTCCCACTGTCCATCTTTCTT
VEGFA-10F	TCGACAGTGAAGCATTCTGGG
VEGFA-10R	CTCCTGGACTGCTATGGTTATG
VEGFA-11F	CTATTTCAGGGACAGGATTGCT
VEGFA-11R	GGACTCTGGCATGACTACTCCA
VEGFA-12F	CTGCTTCCTTCCTTTCCAGATTA
VEGFA-12R	GACTACATCCTCACCTGCATTC
TNFA-1F	CTCTCCATCTGTCAGTCTCATTG
TNFA-1R	AGAGAGACAGGAAGGGAACA
TNFA-2F	CTGTCTCTCTCTCTCTCTCTCTTTG
TNFA-2R	CCCAGAAGGAGGAGGTGTA
TNFA-3F	CTACTTCGTCTACTCCCAGGT
TNFA-3R	TCATGGGCCAGGTAGAGT

TNFA-4F	GAACCATCCCTGATGTCTGTC
TNFA-4R	CTGCTCTTCCTCTGTGTGTG
TNFA-5F	TCAAGTCTTCCCTGATCAAGTC
TNFA-5R	CTCAGCCAGACAGACATCAG
TNFA-6F	CAGAGAGCTTCAGGGATATGTG
TNFA-6R	CCTTCATCTTCTCAGCTTCTCC
TNFA-7F	ACCAGCTAAGAGGGAGAGAA
TNFA-7R	GTATGTGAGAGGAAGAGAACCTG
TNFA-8F	CAGGTTCTCTTCCTCTCACATAC
TNFA-8R	GTCCCGGATCATGCTTTCA
TNFA-9F	GAGACAGAAAGAGCGGGAAATA
TNFA-9R	ATTCACCTTCCAGGCATTCA
TNFA-10F	GCGTGGAGCTGAGAGATAAC
TNFA-10R	TGAAGAGGACCTGGGAGTAG
PAX5-1F	AGAGTCGAGCACTCGGAGAT
PAX5-1R	ACTGAGCAGATTTCGCCACA
PAX5-2F	GCTCGGTCTCTGTGGATCTG
PAX5-2R	GTTCCAGACACCTTACCCCG
PAX5-3F	TTCAAAAACAAAAACCCGGCCT
PAX5-3R	ATTAAGACATGCGCTTGGGG
PAX5-4F	GGGAGCGGAAGGCTTGAAT
PAX5-4R	CTGGTCCGAGGAGTCGGATA
PAX5-5F	TCCGCCAGAGGATAGTGGAA
PAX5-5R	AACCATGGCTGACCCGAAG
PAX5-6F	ATCCAAACCAAAGGTCGCCA
PAX5-6R	ATGGAACTGACGCTAGGCAC
PAX5-7F	GTCTTTCTCCGCGCAGTGTC
PAX5-7R	TTCGTCTCTCTTGCGCTTGT
PAX5-8F	ATTCAGGAGTCTCCGGTGC
PAX5-8R	TGTGTGAACAAGTCTCCCCG
Primers used for genotyping	
H11-F	ATGCCCACCAAAGTCATCAGTGTAG
hPAX5-R	TTCGTCTCTCTTGCGCTTGTTG
hPAX5-F	AAAGTACAGCAGCCACCCAAC
H11-R	TCACAGAAACCATATGGCGCTCC
Trp53$^{fl/fl}$-F	CGCAATCCTTTATTCTGTTCG
Trp53$^{fl/fl}$-R	AGCACATAGGAGGCAGAGAC
Rb1$^{fl/fl}$-F	GGCGTGTGCCATCAATG
Rb1$^{fl/fl}$-R	CTCAAGAGCTCAGACTCATGG
Pax5$^{fl/fl}$-F	AGATAGCCCGCAGCTGTTC
Pax5$^{fl/fl}$-R	TCCACAGCTACTTTGAATAGGG

Supplemental Table 5. Deposited Data.

RNA-seq of H1155 and *PAX5*-KO-H1155 cells	This paper	Accession Number GEO: GSE135439
RNA-seq of H146 and *PAX5*-KD-H146 cells	This paper	Accession Number GEO: GSE280995
RNA-seq of LNCAP and *PAX5*-expressing LNCAP cells	This paper	Accession Number GEO: GSE129887
Microarray of A549 and *PAX5*-expressing A549 cells	This paper	Accession Number GEO: GSE128539
RNA-seq of PC-9 and PAX5-expressing PC-9 cells	This paper	Accession Number GEO: GSE337826
scRNA-seq of Human Primary SCLC	This paper	Accession Number GEO: GSE164145
PAX5-FLAG ChIP-seq	This paper	Accession Number GEO: GSE164913
PAX5 ChIP-seq of H526 cells	This paper	Accession Number GEO: GSE307955
RNA-seq of VEGF treated A549 cells	This paper	Accession Number GEO: GSE307752
