# Supplementary material for: PAX5 modulates vascularization and contributes to neuroendocrine lineage transition during carcinoma progression
Source: J Clin Invest. 2026 Jul 23;136(18):e190596. doi: 10.1172/JCI190596 (PMC13574158; doi:10.1172/JCI190596)

Full unedited blot for Figure 2D

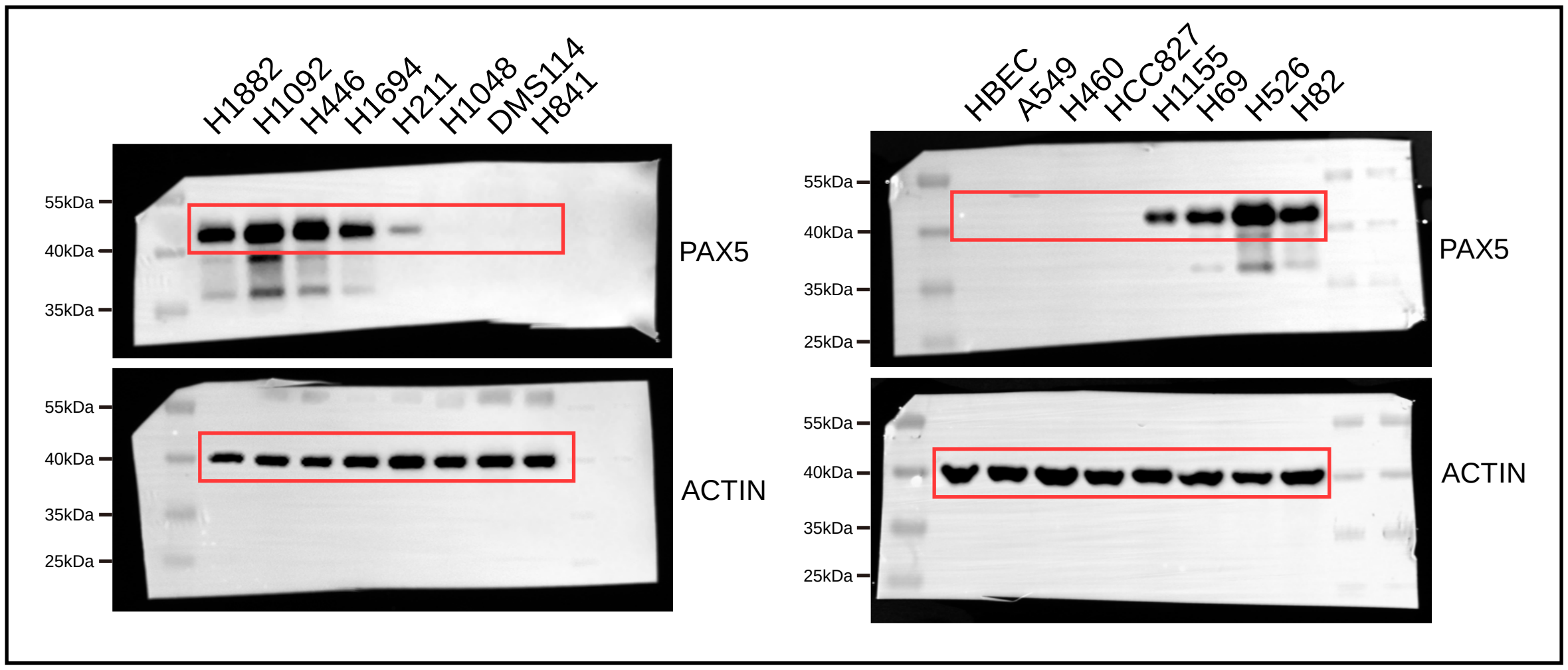

Full unedited blot for Supplemental Figure 4F

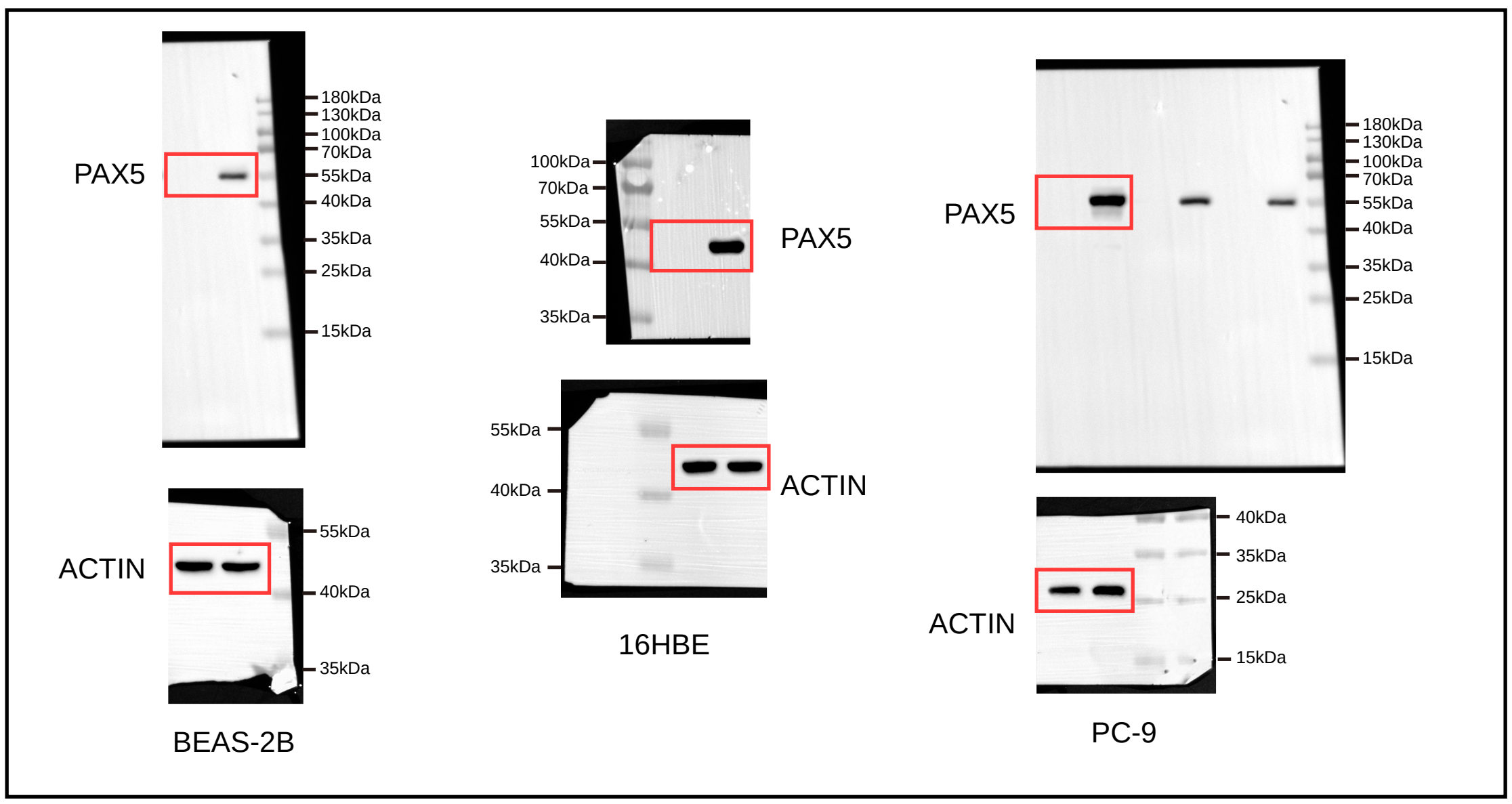

Full unedited blot for Supplemental Figure 6I

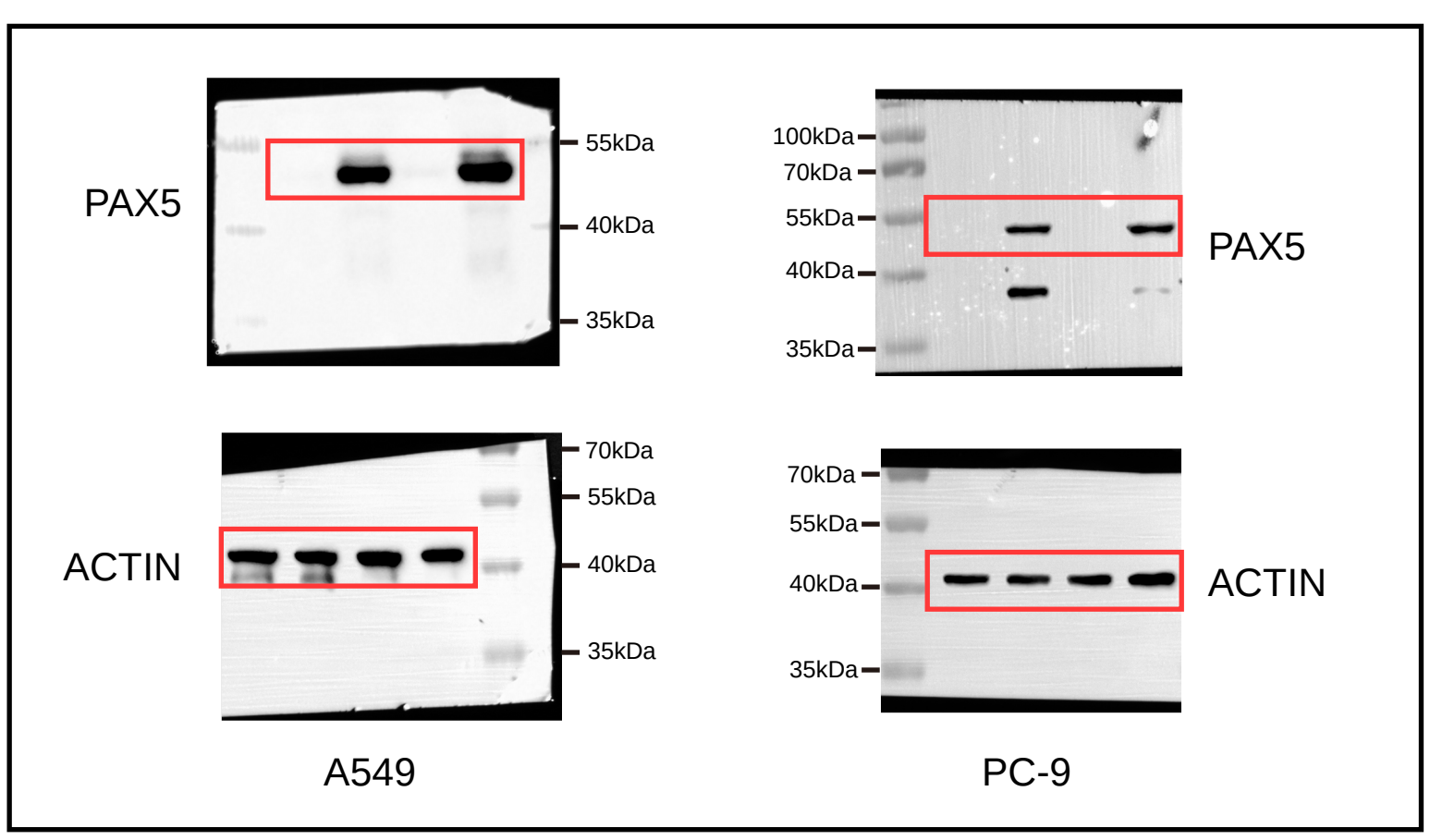

Full unedited blot for Supplemental Figure 8A

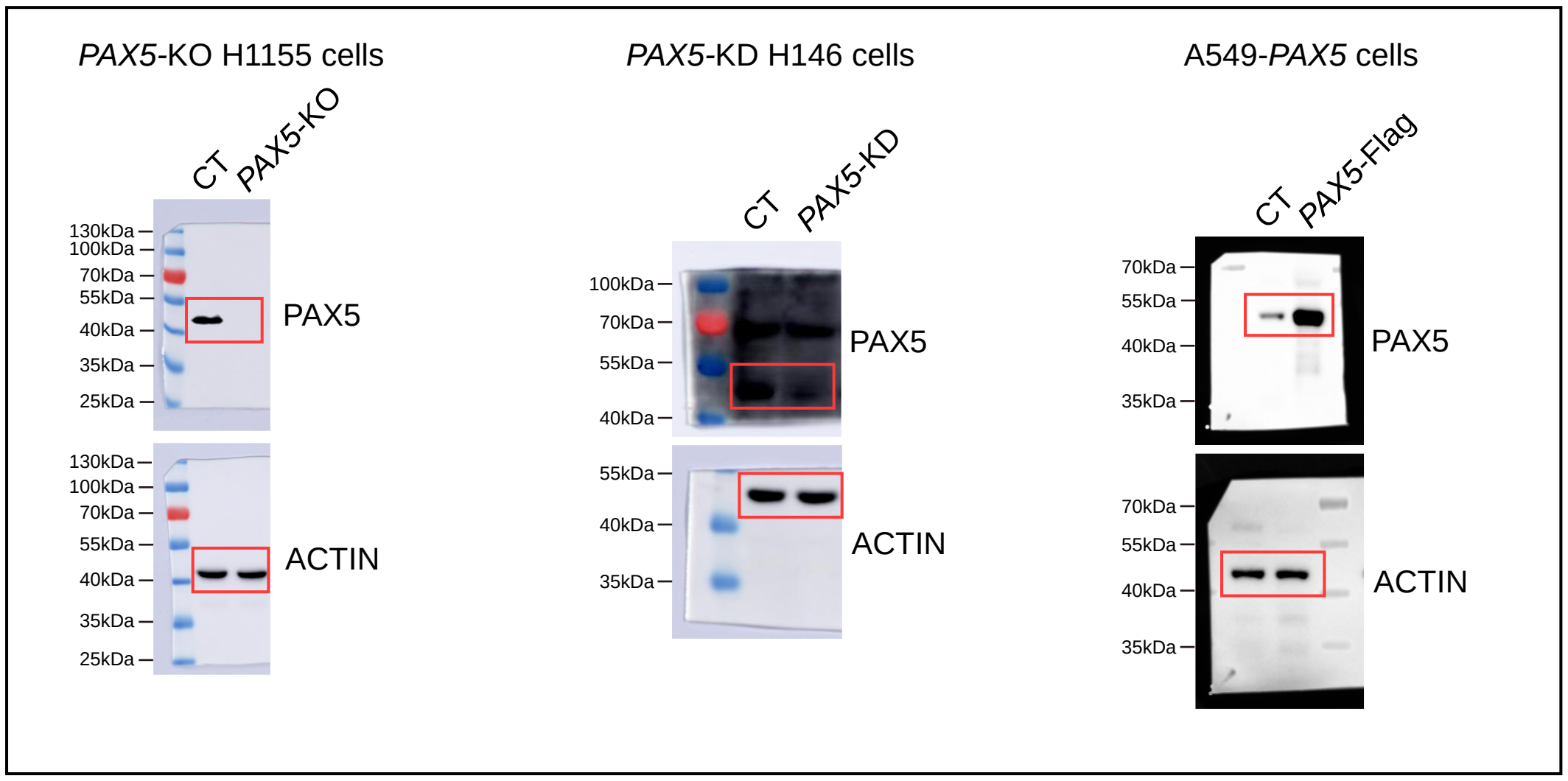

Supplement: Supplementary file 2 [file jci-136-190596-s269.pdf]
